# Roles for H2A.Z and Its Acetylation in *GAL1* Transcription and Gene Induction, but Not *GAL1*-Transcriptional Memory

**DOI:** 10.1371/journal.pbio.1000401

**Published:** 2010-06-22

**Authors:** Jeffrey E. Halley, Tommy Kaplan, Alice Y. Wang, Michael S. Kobor, Jasper Rine

**Affiliations:** 1 Department of Molecular and Cell Biology, and California Institute for Quantitative Biosciences, University of California Berkeley, Berkeley, California, United States; 2 Center for Molecular Medicine and Therapeutics, University of British Columbia, Vancouver, British Columbia, Canada; Adolf Butenandt Institute, Germany

## Abstract

H2A.Z is a histone H2A variant conserved from yeast to humans, and is found at 63% of promoters in *Saccharomyces cerevisiae*. This pattern of localization suggests that H2A.Z is somehow important for gene expression or regulation. H2A.Z can be acetylated at up to four lysine residues on its amino-terminal tail, and acetylated-H2A.Z is enriched in chromatin containing promoters of active genes. We investigated whether H2A.Z's role in *GAL1* gene regulation and gene expression depends on H2A.Z acetylation. Our findings suggested that H2A.Z functioned both in gene regulation and in gene expression and that only its role in gene regulation depended upon its acetylation. Our findings provided an alternate explanation for results that were previously interpreted as evidence that H2A.Z plays a role in *GAL1* transcriptional memory. Additionally, our findings provided new insights into the phenotypes of *htz1*Δ mutants: in the absence of H2A.Z, the SWR1 complex, which deposits H2A.Z into chromatin, was deleterious to the cell, and many of the phenotypes of cells lacking H2A.Z were due to the SWR1 complex's activity rather than to the absence of H2A.Z per se. These results highlight the need to reevaluate all studies on the phenotypes of cells lacking H2A.Z.

## Abstract 2

H2A.Z does not appear to have a role in *GAL1* transcriptional memory, but it does have both acetylation-dependent and acetylation-independent roles in *GAL1* induction and expression.

## Author Summary

Transcriptional memory is the well-documented phenomenon by which cells can “remember” prior transcriptional states. A paradigmatic example of transcriptional memory is found in the yeast *Saccharomyces. S. cerevisiae* remembers prior transcription of the galactose metabolism gene *GAL1*. When a gene is transcribed, the DNA must first be at least partially relieved of its packaging into chromatin by histone proteins. Previous research had suggested that *S. cerevisiae* used a chromatin modification, the incorporation of the histone variant H2A.Z into the region surrounding the *GAL1* promoter, to remember the previous status of *GAL1* transcription. Not all H2A.Z molecules are the same, however. For example, it has recently been discovered that H2A.Z can be acetylated on the four lysine residues in its N-terminal tail region. In an attempt to determine whether H2A.Z acetylation is required for *GAL1* transcriptional memory, we unexpectedly discovered that, although both H2A.Z and H2A.Z acetylation are important for strong and rapid *GAL1* induction, neither H2A.Z nor H2A.Z acetylation plays an important role in *GAL1* transcriptional memory. We propose that the discrepancy between our conclusions and those in prior publications arise from the prior analysis of insufficiently short periods of *GAL1* induction or from complications arising from the comparison of the phenotypes of wild-type yeast strains to those of *htz1*Δ mutants (carrying the null mutation of the gene encoding H2A.Z) mutants. In the current work we show that the *htz1*Δ mutant's phenotype does not simply reflect the absence of H2A.Z in chromatin but instead also reflects the pleiotropic effects of the Swr1 chromatin remodeling complex that is responsible for H2A.Z deposition into chromatin. In the absence of H2A.Z the Swr1 complex itself causes cell damage. In this paper we show that *swr1*Δ *htz1*Δ double mutants have substantially less severe mutant phenotypes than *htz1*Δ mutants. Thus, studies using the *swr1*Δ *htz1*Δ mutant offer more detailed insight into the consequences of the absence of H2A.Z in chromatin than do studies performed on single *htz1*Δ mutants, and our results help to clarify the role of H2A.Z in the regulation of *GAL1* induction and transcriptional memory.

## Introduction

In addition to their role in genome packaging, histones also play a role in the functional organization of eukaryotic genomes. Clear causal relationships have been established between some specific modifications of histones at specific loci and the subsequent events that occur at these loci. Histones are modified by enzymes that couple acetyl, methyl, phosphoryl, ubiquitin, or sumo moieties to specific locations either on histone tails, which extend outward from the nucleosome core, or at positions in the core, such as acetylation of H3 lysine 56, near where the DNA helix enters and leaves the nucleosome [1]. Modified histone tails serve in some cases as docking sites for protein complexes. Thus, in principle, a particular collection of modifications on the nucleosomes of a locus can recruit specific complexes to that locus to achieve a particular outcome [2]–[7].

In addition to histone modifications, nucleosomes can also be specialized by virtue of the presence of histone variants. *Saccharomyces* encodes three histone variants: H2A.Z, which is conserved from yeast to humans; a variant of H2B called H2B2, conserved among yeasts; and Cse4, an H3 variant, which functions at the nucleosomes at centromeres [8].

Like Cse4p, H2A.Z is also localized to specific chromosomal locations with specialized functions. In *S. cerevisiae*, H2A.Z is incorporated into nucleosomes near, but not at, centromeres, at the borders of heterochromatic domains, and near the promoters of 63% of genes [9]–[12]. H2A.Z is incorporated into chromatin by the SWR1 complex (SWR1-Com) a multi-subunit enzyme whose catalytic subunit, Swr1, is a member of the Swi2/Snf2 family of chromatin remodeling enzymes [13]–[15].

H2A.Z's localization at promoters suggests that it plays an important role in gene expression. Yet genome-wide micro-array analyses indicate that H2A.Z affects the steady-state mRNA levels of only 5% of *S. cerevisiae's* genes [16]. Interestingly, most of the genes downregulated in cells lacking H2A.Z were near the boundaries of SIR-silenced heterochromatin. This observation revealed that H2A.Z functions as part of the boundary separating euchromatin and heterochromatin [16].

H2A.Z is acetylated at up to four positions on its N-terminal tail by the NuA4 and SAGA histone-acetyltransferase complexes [17]–[19]. Moreover, H2A.Z's heterochromatin-boundary function depends on its acetylation [17]. Promoter-proximal H2A.Z is also acetylated and, as measured on a cell population, the level of acetylation correlates with the gene's expression level [19]. Recent work suggests that acetylated-H2A.Z promotes transcription of adjacent genes. Specifically, H2A.Z at the promoters of the oleate-responsive genes *CTA1*, *POX1*, *POT1*, and *FOX2* is acetylated on Lys14. Cells with a mutant form of H2A.Z that cannot be acetylated at this position are defective in induction of these genes [20].

H2A.Z's contribution to gene-induction was first explored in the context of the *GAL1*, *GAL7*, and *GAL10* genes [21],[22], which are induced in medium containing galactose, repressed in medium containing glucose, and expressed at a basal uninduced level by cells grown in medium with nonfermentable carbon sources [23]–[26]. Because the galactose regulon is one of only a handful of thoroughly studied regulated genes in yeast, it has provided many fresh insights into gene regulation. Hence results on, and claims about, this regulon take on special importance in the field.

Induction of *GAL1*, *GAL7*, and *GAL10* occurs more rapidly when *S. cerevisiae* cells are grown in a nonrepressing, noninducing carbon source (such as raffinose) and then shifted to inducing conditions (galactose) than when cells are grown in repressing conditions (glucose) and then transferred into inducing conditions [23]–[26]. The one exception to this pattern involves a phenomenon known as transcriptional memory. *S. cerevisiae* cells grown in inducing conditions prior to short-term growth in repressing conditions are able to reinduce *GAL*- gene expression upon induction as rapidly as cells grown continuously in nonrepressing conditions [27]–[29]. This “memory” of recent inducing conditions is reported to be H2A.Z dependent [27], although other explanations have been offered [29].

The role of H2A.Z in galactose induction extends beyond its role in *GAL1* transcriptional memory. Cells that are grown in nonrepressing conditions prior to galactose induction require H2A.Z for the rapid induction of *GAL1* [21],[22]. H2A.Z promotes the rapid induction of *GAL1* by recruiting the Mediator complex to the *GAL1* promoter [30],[31].

The work presented in this paper was aimed at testing the potential role of H2A.Z acetylation in gene induction and transcriptional memory. We found no evidence for a role for H2A.Z in *GAL1* transcriptional memory, discovered a role for H2A.Z acetylation in gene induction, and discovered a confounding influence of SWR1-Com on gene regulation in cells lacking H2A.Z.

## Results

### Acetylated H2A.Z Was Important for Primary Induction of GAL1 Transcription but Did Not Play a Specialized Role in GAL1-transcriptional Memory

Upon galactose induction, cells previously grown long-term in repressing conditions induce *GAL1* expression more slowly than cells previously grown in noninducing-nonrepressing conditions. The conclusion that H2A.Z is essential for *GAL1* transcriptional memory was based on the following two observations. First, when transferred to inducing conditions from long-term growth in repressing conditions *HTZ1* and *htz1*Δ cells induce *GAL1* slowly and at a similar rate [27]. Second, when transferred to inducing conditions from short-term growth in repressing conditions, *HTZ1* cells induce *GAL1* transcription rapidly, but *htz1*Δ cells are reported to not induce *GAL1* any more rapidly than *htz1*Δ cells that had been grown long term in repressing conditions prior to galactose induction [27]. We reasoned that if H2A.Z acetylation were required exclusively for transcriptional memory, then cells carrying an unacetylatable allele of *HTZ1*, *htz1-K3,8,10,14R*, would exhibit defective *GAL1* induction following short-term growth in glucose, but exhibit normal *GAL1* induction following long-term growth in glucose.

**Figure 1:**
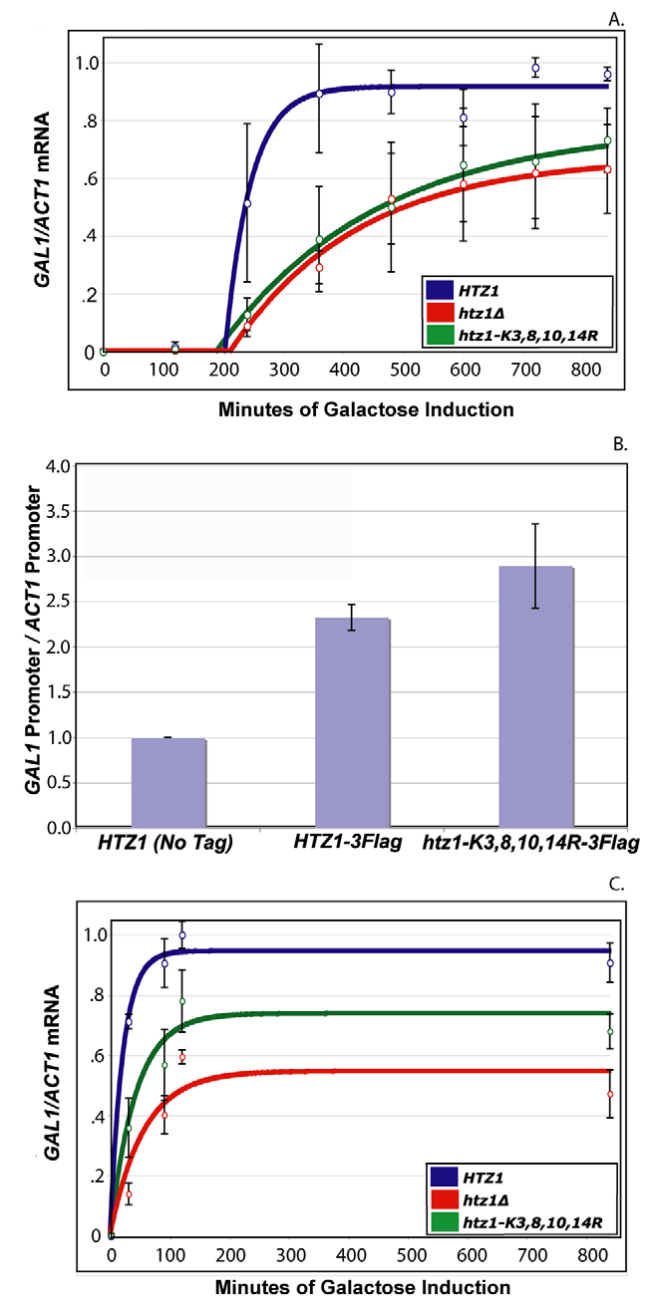
Acetylated H2A.Z was important for *GAL1* induction. Q-RT PCR of *GAL1* mRNA performed on *HTZ1* (JRY7971), *htz1*Δ (JRY7754), and *htz1-K3,8,10,14R* (JRY7983) cultures that were grown long-term in YP-glucose (2%) prior to being transferred into YP-galactose (2%). Open circles represent the average of three biological replicates. Bars represent standard deviations of values from these replicates. Solid lines represent the best-fit curve for the measured data. See text for details. (B) ChIP analysis of H2A.Z-FLAG at the *GAL1* promoter in cells grown long-term in YP-glucose (2%). (C) Q-RT PCR of *GAL1* mRNA performed on *HTZ1* (JRY7971), *htz1*Δ (JRY7754), and *htz1-K3,8,10,14R* (JRY7983) cultures that were grown for 20 h in YP-galactose (2%) prior to 12 h of growth in YP-glucose (2%) prior to being transferred into YP-galactose (2%). Open circles represent the average of three biological replicates. Bars represent standard deviations of values from these replicates. Solid lines represent the best-fit curve for the measured data. See text for details.

To determine first whether H2A.Z-acetylation had any role in galactose expression, *GAL1* mRNA levels were evaluated by quantitative reverse transcriptase (Q-RT) PCR in *HTZ1* (JRY7971), *htz1*Δ (JRY9001), and *htz1-K3,8,10,14R* (JRY7983) cultures grown in long-term repressing conditions prior to galactose induction. Cells grown continuously in glucose medium were transferred to galactose medium and *GAL1* induction was evaluated at 2-h intervals for 14 h. One characteristic shared between all three strains' *GAL1* induction phenotypes was an approximately 3-h lag period with little to no *GAL1* expression. Quantitative analysis suggested that neither *htz1*Δ nor *htz1-K3,8,10,14R* cultures exhibited substantially different lag periods prior to the onset of *GAL1* expression than those exhibited by *HTZ1* cultures (Figure 1A; Table 1, column A). These results suggested that neither H2A.Z nor its acetylation influenced how rapidly the cultures exited glucose repression and began *GAL1* transcription.

**Table 1:** Quantitative analysis of *GAL1* transcription phenotypes.

Column	A	B	C	D	E	F	G	H
Strain	Primary Induction *GAL1* Transcription Activation Time (min)	Secondary Induction *GAL1* Transcription Activation Time (min)	Primary Induction *GAL1* Steady State Expression Level (*GAL1/ACT1*)	Secondary Induction *GAL1* Steady State Expression Level (*GAL1/ACT1*)	Primary Induction Time to Half-Steady-State *GAL1* Expression Level (min)	Secondary Induction Time to Half-Steady-State *GAL1* Expression Level (min)	Primary Induction Time from Activation to Half- Steady-State Levels (min)	Secondary Induction Time from Activation to Half- Steady-State Levels (min)
***HTZ1***	204.8	0^a^	0.9	0.9	234.3	14.8	29.5	14.8
***htz1***Δ	214.1	0^a^	0.7	0.6	362.4	38.9	148.3	38.9
***htz1-K3,8,10,14R***	191.3	0^a^	0.8	0.7	375.4	29.7	184.2	29.7
***HTZ1*** ** (CRY1)**	204.4	0^a^	0.8	1.0	243.7	28.4	39.3	28.4
***htz1*** **Δ (DBY 50)**	218.9	0^a^	0.7	0.7	356.3	110.4	137.4	110.4

a The data suggested that the time to first induction of *GAL1* for all strains in the secondary induction experiments was very close to zero, and could not be distinguished from it.

Other than their lag periods the two mutant cultures exhibited significantly different *GAL1* induction phenotypes than those of *HTZ1* cultures. Cultures of the two mutant strains had lower steady-state *GAL1* expression levels than *HTZ1* cultures (Figure 1A; Table 1, column C). Quantitative analysis suggested that *htz1*Δ and *htz1-K3,8,10,14R* cultures required 54.7% and 60.2% more time, respectively, than *HTZ1* cultures to reach half steady-state *GAL1* expression levels (Figure 1A; Table 2, column E; note that half steady-state levels were used instead of half-maximum levels because the level of expression during induction typically overshot the induced steady-state level). These values, however, underplayed the severity of the *htz1*Δ and *htz1-K3,8,10,14R* cultures' *GAL1*-transcription rate phenotypes because all three strains spent the majority of time that was required to reach half-steady-state levels in the lag period prior to *GAL1* activation (Figure 1A; Table 1, columns A and E; note that half steady-state levels were used instead of half-maximum levels because the level of expression during induction typically overshot the induced steady-state level). To accurately compare the *GAL1* transcription rates of the three strains it was necessary to determine the amount of time that cultures of these strains required to reach half-steady-state levels of *GAL1* expression from the time of *GAL1* activation. These values were determined for each culture by subtracting its *GAL1* activation time from the time required to reach the half steady-state level of *GAL1* expression. This analysis revealed that once they had begun expressing *GAL1*, *htz1*Δ and *htz1-K3,8,10,14R* cultures required 503% and 625% of the time required for *HTZ1* cultures, respectively, to express *GAL1* at half-steady-state levels (Table 2, column G). Thus, both H2A.Z and its acetylation contributed to the rate of *GAL1* expression in cultures grown under long-term glucose repression prior to galactose induction.

**Table 2:** *GAL1* induction phenotypes relative to *HTZ1* phenotypes (percent of *HTZ1* values).

Column	A	B	C	D	E	F	G	H
Strain	Primary Induction *GAL1* Transcription Activation Time (min)	Secondary Induction *GAL1* Transcription Activation Time (min)	Primary Induction *GAL1* Steady State Expression Level (*GAL1/ACT1*)	Secondary Induction *GAL1* Steady State Expression Level (*GAL1/ACT1*)	Primary Induction Time to Half-Steady-State *GAL1* Expression Level (min)	Secondary Induction Time to Half-Steady-State *GAL1* Expression Level (min)	Primary Induction Time from Activation to Half- Steady-State Levels (min)	Secondary Induction Time from Activation to Half- Steady-State Levels (min)
***HTZ1***	100.0	100.0	100.0	100.0	100.0	100.0	100.0	100.0
***htz1*** **Δ**	95.7	100.0	73.2	57.7	154.7	262.5	503.5	262.5
***htz1-K3,8,10,14R***	107.1	100.0	84.6	78.0	160.2	200.4	625.3	200.4
***HTZ1*** ** (CRY1)**	100.0	100.0	100.0	100.0	100.0	100.0	100.0	100.0
***htz1*** **Δ (DBY 50)**	107.0	100.0	79.5	73.0	146.2	389.0	349.4	389.0

Because the expression of *GAL1* in *htz1*Δ and *htz1-K3,8,10,14R* strains was similar, the role of H2A.Z in *GAL1* expression was presumably dependent upon its acetylation. To determine whether H2A.Z acetylation affected the level of H2A.Z at the *GAL1* promoter, chromatin immunoprecipitation experiments were performed with qPCR to quantitate the level of enrichment. Both acetylatable and unacetylatable H2A.Z were present at approximately equal levels at *GAL1* (Figure 1b). Therefore, acetylation of H2A.Z was important for *GAL1* induction at some point after H2A.Z's incorporation at the *GAL1* promoter.

To determine whether H2A.Z acetylation had a role in transcriptional memory, *GAL1* mRNA levels were evaluated in *HTZ1* (JRY7971), *htz1*Δ (JRY9001), and *htz1-K3,8,10,14R* (JRY7983) cultures that were grown short-term in repressing conditions prior to galactose induction. Cells grown in galactose medium prior to short-term growth in glucose medium (12 h) were transferred to galactose medium and *GAL1* induction was evaluated for 14 h in inducing conditions. None of the three strains exhibited a significant lag in *GAL1* expression (Figure 1C; Table 1, column B). Quantitative analysis of these data suggested that all three strains, when grown short-term in repressing conditions, expressed *GAL1* in half the time, or less, than when the same strains were induced following long-term growth in repressing conditions (Table 3, column D). The combined effect of near-zero onset times and increased *GAL1* transcription rates was that all three strains reached half steady-state *GAL1* expression levels in 90% less time than was required for the same strains to reach this level when they were grown long-term in repressing conditions prior to galactose induction (Table 3, column C). Thus, all three strains exhibited transcriptional memory with respect to *GAL1* transcription. Importantly, relative to the *HTZ1* strain, the two mutant strains exhibited less severe phenotypes when they were grown short-term in repressing conditions prior to induction than when they were grown long term in repressing conditions prior to induction (Table 2, compare column G with H). Thus, neither H2A.Z nor its acetylation played an important role in *GAL1*-transcriptional memory.

**Table 3:** Percent change in *GAL1* induction phenotypes between primary and secondary inductions.

Column	*A*	*B*	*C*	*D*
Strain	*GAL1* Transcription Activation Time	*GAL1* Steady State Expression Level	Time to Half-Max Steady-State *GAL1* Expression Level	Time from Activation to Half-Max Steady-State Levels
***HTZ1***	−100.0	3.3	−93.7	−49.8
***htz1Δ***	−100.0	−17.9	−89.3	−73.8
***htz1-K3,8,10,14R***	−100.0	−3.9	−92.1	−83.9
***HTZ1*** ** (CRY1)**	−100.0	15.5	−88.4	−27.7
***htz1*** **Δ (DBY 50)**	−100.0	6.0	−69.0	−19.7

**Figure 2:**
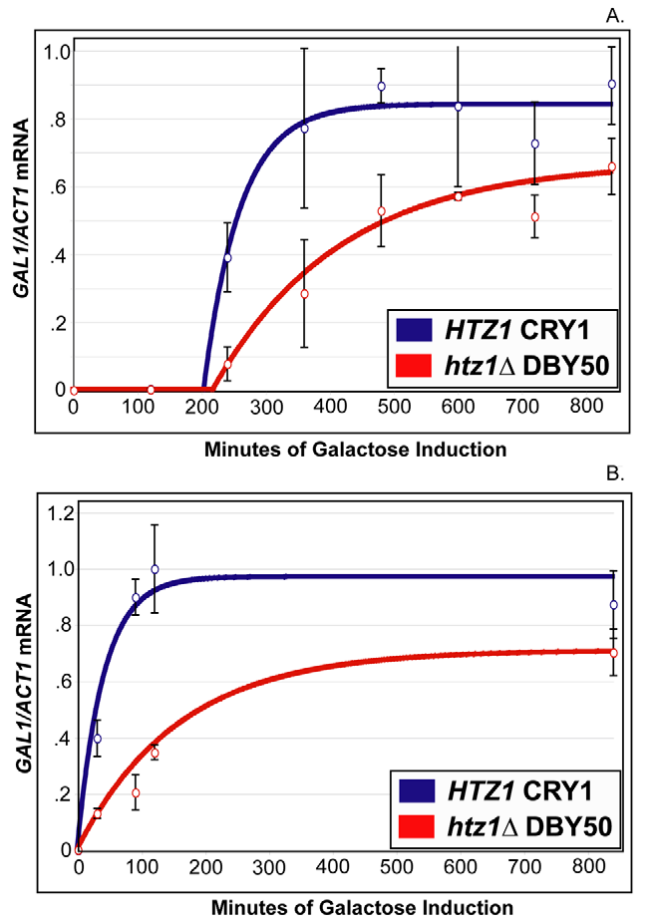
H2A.Z was not required for *GAL1* transcriptional memory. (A) Q-RT PCR of *GAL1* mRNA performed on RNA from *HTZ1* (CRY1) and *htz1* (DBY50) grown long term in CSM-Glucose (2%) prior to being transferred into CSM galactose (2%). Open circles represent the average of three biological replicates. Bars represent standard deviations of values from these replicates. Solid lines represent the best-fit curve for the measured data. See text for details. (B) Q-RT PCR of *GAL1* mRNA performed on RNA from *HTZ1* (CRY1) and *htz1* (DBY50) grown in CSM-galactose (2%) for 20 h prior to being grown in CSM-Glucose (2%) for 12 h prior to being transferred into CSM galactose (2%). Open circles represent the average of two biological replicates. Bars represent standard deviations of values from these replicates. Solid lines represent the best-fit curve for the measured data. See text for details.

Because the results described above differed substantially from ostensibly equivalent experiments [27], we obtained the strains used in the previously published experiments, *HTZ1* (CRY 1) and *htz1*Δ (DBY 50), and attempted to reproduce the previously published results. Just as described above, both *HTZ1* (CRY 1) and *htz1*Δ (DBY 50) cultures exhibited a similar lag period before *GAL1* mRNA was detectable (Figure 2A; Table 1, column A). As before, when grown under long-term repressing conditions prior to galactose induction, galactose-induced *HTZ1* (CRY1) cells had both higher steady-state *GAL1* mRNA levels and faster *GAL1* transcription rates than *htz1*Δ (DBY 50) cells (Figure 2A; Table 1, columns D and G). Quantitative analysis suggested that once both cultures had begun expressing *GAL1*, the *htz1*Δ (DBY 50) cultures required about 3.5× more time than *HTZ1* (CRY 1) cultures to reach half-steady-state *GAL1* expression levels (Table 2, column G). Additionally, as was the case with the other set of strains, both *HTZ1* (CRY 1) and *htz1*Δ (DBY 50) cultures induced *GAL1* expression significantly more rapidly when grown short-term (12 h) in repressing conditions prior to galactose induction than when the same cultures were grown long term in repressing conditions prior to galactose induction (Figure 2B; Table 1, column B). Cultures of both strains also required significantly less time to accumulate half-steady state levels of *GAL1* mRNA when grown short term rather than long term in repressing conditions prior to galactose induction: *HTZ1* (CRY 1) and *htz1*Δ (DBY 50) cultures required 88% and 69% less time, respectively, under these conditions to accumulate half-steady levels of *GAL1* mRNA transcripts (Table 3, column C). Thus, as before, both *HTZ1* and *htz1*Δ cultures exhibited transcriptional memory of prior *GAL1* induction. Therefore, H2A.Z was important for *GAL1* induction regardless of whether cells were induced from short-term or long-term growth in repressing conditions prior to induction.

### Acetylated-H2A.Z Allowed Cells to Activate *GAL1* Expression Efficiently

Two factors contribute to the *GAL1* expression level in a culture of cells: the proportion of cells that are expressing *GAL1*, and the level of *GAL1* expression in the fraction of cells in which it is expressed. *S. cerevisiae* regulates *GAL1* expression in response to different growth conditions both by increasing the number of *GAL1*-expressing cells and by increasing the level of *GAL1* expression. Both parameters respond independently to different aspects of growth conditions [32].

**Figure 3:**
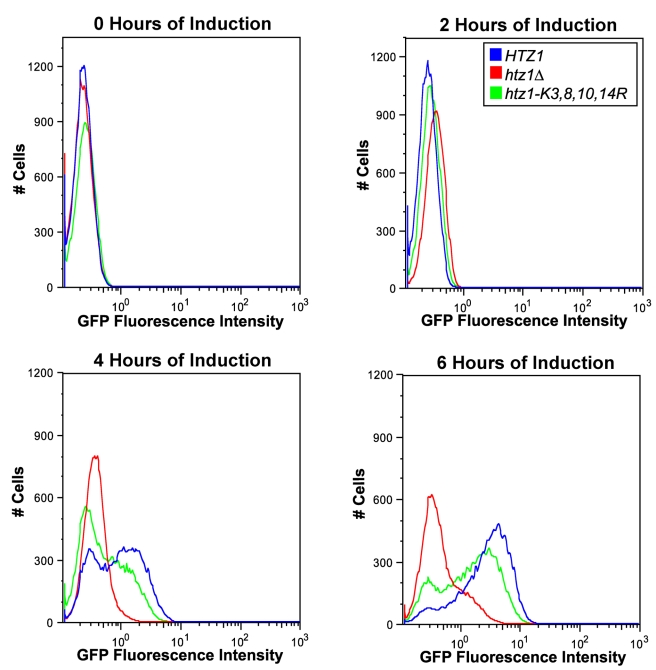
Acetylated H2A.Z was important for *GAL1* gene induction. Flow cytometry analysis was performed using Gal1-GFP on *HTZ1* (JRY9002), *htz1*Δ (JRY9004), and *htz1-K3,8,10,14R* (JRY9003) cells grown long-term in YP-glucose (2%) prior to being transferred into YP-galactose (2%). The histograms in this figure represent the distribution of cells within each culture as a function of their GFP intensity.

To determine whether *htz1*Δ and *htz1-K3,8,10,14R* cultures' *GAL1*- expression defects were attributable to decreased proportions of *GAL1*-expressing cells, or to decreased *GAL1* expression level per cell, flow cytometry was used to monitor galactose induction of a fusion protein containing the entire *GAL1* coding sequence, with a C-terminal fusion to green fluorescent protein (GFP), in *htz1-K3,8,10,14R*, *htz1*Δ, and *HTZ1* cells (Figures 3 and S6, S7, S8).

If H2A.Z were to contribute to the probability that a cell enters the galactose-induced state per unit of time, but not to the expression level in those induced cells, then *htz1*Δ cultures should have a smaller proportion of GFP-positive cells at each postinduction time point than *HTZ1* cultures, but the GFP-positive cells should have similar fluorescence intensities to those in *HTZ1* cultures. However, if H2A.Z were important for achieving high expression levels but did not influence the probability of induction per se, then *htz1*Δ and *HTZ1* cultures should have similar proportions of GFP-positive cells, but the *GAL1*-*GFP*-expressing cells from *htz1*Δ mutant cultures would have lower GFP fluorescence than *GAL1-GFP*-expressing cells from *HTZ1* cultures. The same logic would apply to the possible roles of H2A.Z acetylation.

**Figure 4:**
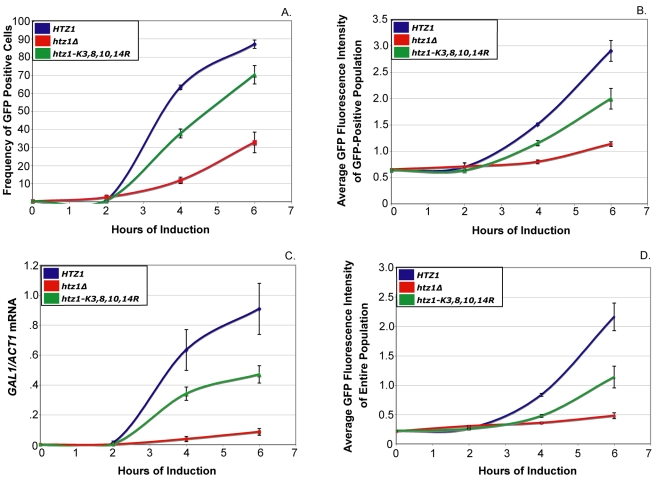
*htz1*Δ cells' galactose induction phenotypes are more severe than those of *htz1-K3,8,10,14R* cells. A threshold level of GFP-intensity was set so that between 1% and 2% of glucose-grown *HTZ1* cultures were classified as GFP-positive cells. (A) The frequency of GFP-positive cells within *HTZ1*, *htz1*Δ, and *htz1-K3,8,10,14R* cultures. (B) The average GFP intensity of the GFP-positive populations of *HTZ1*, *htz1*Δ, and *htz1-K3,8,10,14R* cultures. (C) Q-RT PCR of *GAL1-GFP* mRNA performed on *HTZ1* (JRY9002), *htz1*Δ (JRY9004), and *htz1-K3,8,10,14R* (JRY9003) cultures that were grown long-term in YP-glucose (2%) prior to being transferred into YP-galactose (2%). (D) The average GFP intensity of the entire population of cells, both GFP positive and negative, within *HTZ1, htz1*Δ, and *htz1-K3,8,10,14R* cultures. Bars in all panels represent the standard deviations of values from three biological replicates.

To compare the results of these experiments, a threshold value of GFP-intensity was used to classify cells as either GFP-positive or GFP-negative. This threshold was set so that between 1% and 2% of cells from noninduced *HTZ1* cultures were classified as GFP-positive. On average *htz1-K3,8,10,14R* cultures had 33% fewer GFP-positive cells than *HTZ1* cultures at all postinduction time points (Figures 3 and 4A). Additionally, GFP-positive cells from *htz1-K3,8,10,14R* cells had, on average, 17% lower mean-GFP intensity than *HTZ1* cultures (Figures 3 and 4B).

**Figure 5:**
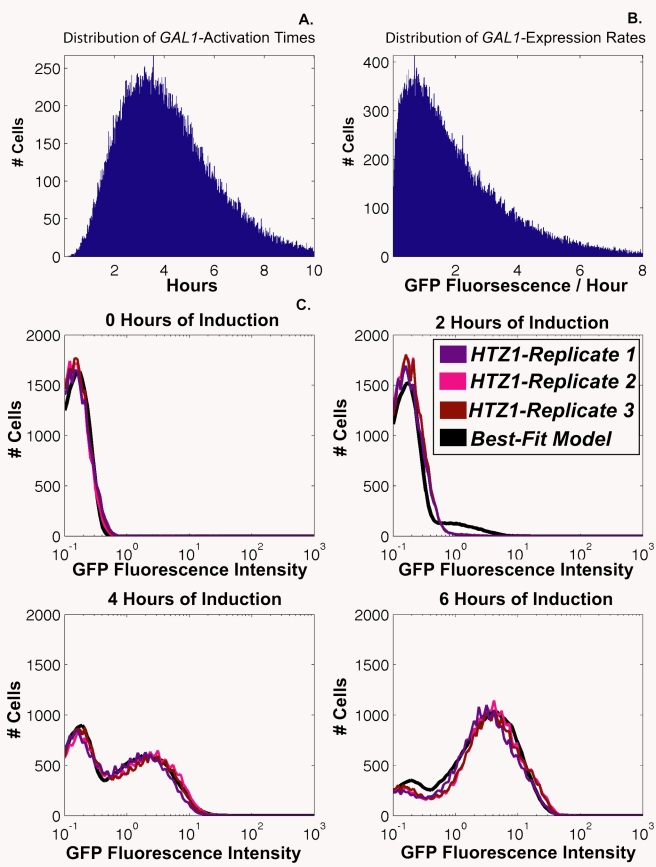
The distribution of *GAL1*-induction times and Gal-GFPp accumulation rates among cells as modeled as a Gamma distribution of values. See text for details. (A) shows the Gamma distribution of *GAL1*-induction times that were used in the best-fit simulation of *HTZ1's* (JRY9002) *GAL1-GFP* expression phenotype. (B) shows the Gamma distribution of Gal1-GFP accumulation rates that were used in the best-fit simulation of *HTZ1's* (JRY9002) *GAL1-GFP* expression phenotype. (C) compares the *GAL1-GFP* induction phenotypes observed for *HTZ1* cultures with the phenotype predicted for *HTZ1* based on its best-fit simulation.

The simplest interpretation of these findings was that H2A.Z-acetylation influenced both the time required to induce *GAL1-GFP* expression and the rate at which Gal1-GFP accumulated once induced. Another possibility was that the differences between *HTZ1* and *htz1-K3,8,10,14R* cells were due exclusively to differences in either the time required for induction or to the rate of Gal1-GFP accumulation. To distinguish between these two possibilities, the *GAL1*-induction times and Gal1-GFP accumulation rates were determined for both cultures by fitting a simple mathematical model of gene expression to the data for each culture (the model is described in Materials and Methods; Figures 5 and S1, S2, S3, S4). The model simulated the galactose induction phenotype of a culture by estimating the distribution of activation times and expression rates of the measured cells. The model's parameters were fitted to the observed data for each strain by optimizing the fit to cell-specific measurements of *GAL1-GFP* levels. Each culture's average induction time and average accumulation rate are presented in Tables 4 and 5, respectively.

**Table 4:** *GAL1*-activation times.

Strain	Mean Time of Activation (h)	Percent Difference From *HTZ1*
***HTZ1***	4.2	0.0
***htz1*** **Δ**	6.5	54.6
***htz1-k3,8,10,14R***	5.5	31.2
***swr1*** **Δ ** ***HTZ1***	5.2	25.2
***swr1*** **Δ ** ***htz1*** **Δ**	5.3	27.6

**Table 5:** *GAL1*-expression rates.

Strain	Mean Expression Rate (GFP Counts/h)	Percent Difference From *HTZ1*
***HTZ1***	2.2	0.0
***htz1*** **Δ**	1.3	−38.8
***htz1-k3,8,10,14R***	2.1	−2.4
***swr1*** **Δ ** ***HTZ1***	1.6	−24.0
***swr1*** **Δ ** ***htz1*** **Δ**	1.6	−26.1

This analysis revealed that *htz1-K3,8,10,14R* cells induced *GAL1-GFP* expression 31% (+/−3.3%) more slowly than did *HTZ1* cells (Table 4), and that induced cells in both *HTZ1* and *htz1-K3,8,10,14R* cultures accumulated Gal1-GFP at similar rates (Table 5). Thus, with respect to *GAL1* induction, H2A.Z-acetylation reduced the amount of time required to induce *GAL1*, but did not influence the rate at which induced cells accumulated Gal1-GFPp.

Interestingly, *htz1*Δ cells had a more severe defect in *GAL1*-GFP expression phenotypes than did cells with unacetylatable H2A.Z (Figures 3, 4A, and 4B; Tables 4 and 5). On average, *htz1*Δ cultures had 28% fewer GFP-positive cells than *htz1-K3,8,10,14R* cultures at the 4-h and 6-h time points. At these same time points, the average GFP-intensity of GFP-positive cells in *htz1*Δ cultures was 46% lower than that of GFP-positive cells in *htz1-K3,8,10,14R* cultures. Moreover, *htz1*Δ cells induced *GAL1-GFP* 18.2% (+/−3.8%) later and accumulated Gal1-GFP 38.1% (+/−5.6%) more slowly than *htz1-K3,8,10,14R* cells (Tables 4 and 5). These results were surprising because *htz1Δ* and *htz1-K3,8,10,14R* cultures had similar *GAL1* mRNA induction phenotypes (Figure 1A). mRNA analysis revealed that the *htz1*Δ cultures used in these experiments accumulated *GAL1-GFP* transcripts, in contrasts to the *GAL1* transcripts in Figure 1A, more slowly than either *HTZ1* or *htz1-K3,8,10,14R* culture (Figure 4C). These results suggested that *htz1Δ* cells accumulated Gal1-GFP more slowly than *htz1-K3,8,10,14R* cells because they produced *GAL1-GFP* mRNA more slowly than *htz1-K3,8,10,14R* cells.

All of the mRNA measurements performed in this study were performed on bulk cultures, whereas the flow cytometry measurements were made on single cells within cultures. To determine whether the flow cytometry measurements of Gal1-GFP accumulation in *HTZ1*, *htz1*Δ, and *htz1-K3,8,10,14R* strains corresponded well with each strain's *GAL1-GFP* mRNA accumulation phenotype, the average GFP intensity of each culture was determined (Figure 4D). The galactose-induction phenotypes of all three strains, as measured by average GFP accumulation, were qualitatively similar to their galactose induction phenotypes as measured by *GAL1-GFP* mRNA accumulation. Thus, the flow-cytometry data in these studies reflected *GAL1-GFP* mRNA accumulation.

At face value, the more severe galactose-induction phenotypes of *htz1*Δ than of *htz1-K3,8,10,14R* cells suggested that H2A.Z's role in *GAL1* induction was only partially dependent on its acetylation. However, as described below, the more severe *GAL1*-expression defects in *htz1*Δ cells resulted from secondary complications that arose from the action of SWR1-Com in cells lacking H2A.Z.

### Overlapping Contribution of Individual H2A.Z Acetylation Sites to *GAL1* Induction

**Figure 6:**
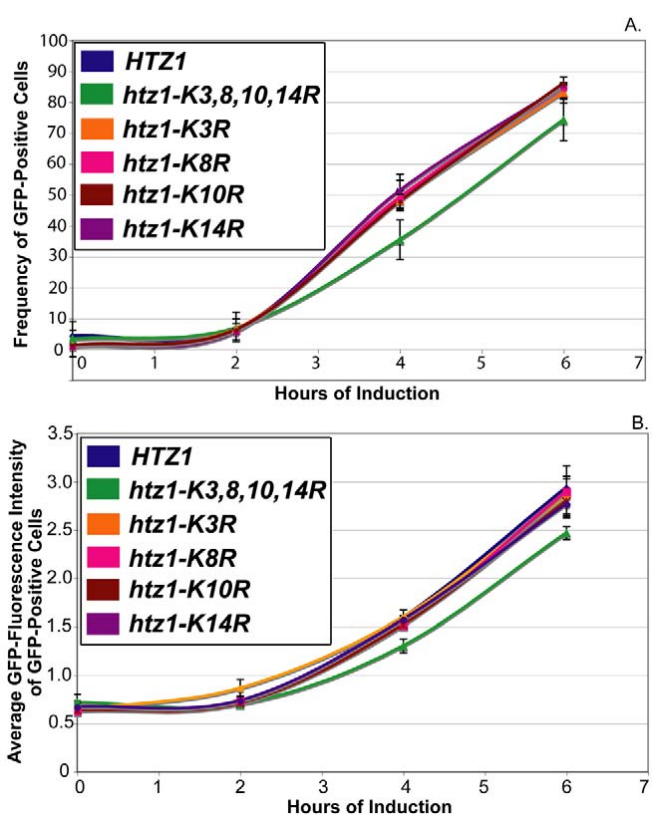
Role of H2A.Z acetylation in *GAL1* induction. Flow cytometry analysis was performed using Gal1-GFP on *HTZ1/htz1*Δ (JRY9007), *htz1-K3,8,10,14R/htz1*Δ (JRY9008), *htz1-K3R/htz1*Δ (JRY9009), *htz1-K8R/htz1*Δ (JRY2010), *htz1-K10R/htz1*Δ (JRY2011), and *htz1-K14R*/*htz1*Δ (JRY2012) cells grown long-term in YP-glucose (2%) prior to being transferred into YP-galactose (2%). A threshold level of GFP-intensity was set so that between 1% and 2% of glucose-grown *HTZ1* cultures were classified as GFP-positive cells. (A) The frequency of GFP-positive cells within *HTZ1*, *htz1-K3,8,10,14R*, *htz1-K3R*, *htz1-K8R*, *htz1-K10R*, and *htz1-K14R* cultures. (B) The average GFP intensity of the GFP-positive populations of *HTZ1*, *htz1-K3,8,10,14R*, *htz1-K3R*, *htz1-K8R*, *htz1-K10R*, and *htz1-K14R* cultures. Bars in all panels represent the standard deviations of values from three biological replicates.

Acetylation of lys14 on H2A.Z is important for its role in *FOX2* and *POT1* induction [20]. To determine whether the acetylation of lys14 or other lysine residues of H2A.Z contributed to *GAL1* induction, the *GAL1* induction phenotypes of diploid cultures each with one null allele and individual lys-to-arg mutations as the other allele (*htz1-K3R/htz1*Δ, *htz1-K8R/htz1*Δ, *htz1-K10R/htz1*Δ, and *htz1-K14R/htz1*Δ) were determined using flow-cytometry. Surprisingly, none of the single acetylation-site mutants exhibited *GAL1-GFP* expression defects (Figure 6; example FACS profiles are in Figures. S6, S7, S8). Thus, H2A.Z's role in *GAL1* induction depended on its acetylation, but did not depend exclusively on the acetylation of any single tail-lysine residue. These results were surprising given the focus on the acetylation of H2A.Z lys14 in previous studies in *S. cerevisiae* [18],[19], but they are consistent with discoveries made in *Tetrahymena*. In *Tetrahymena*, acetylation of H2A.Z's tail lysines contributes to H2A.Z's function simply by decreasing the positive charge of H2A.Z's tail and thus all sites of acetylation function equally well in this respect [33].

### SWR1-Com Enhanced Many *htz1Δ* Mutant Phenotypes

**Figure 7:**
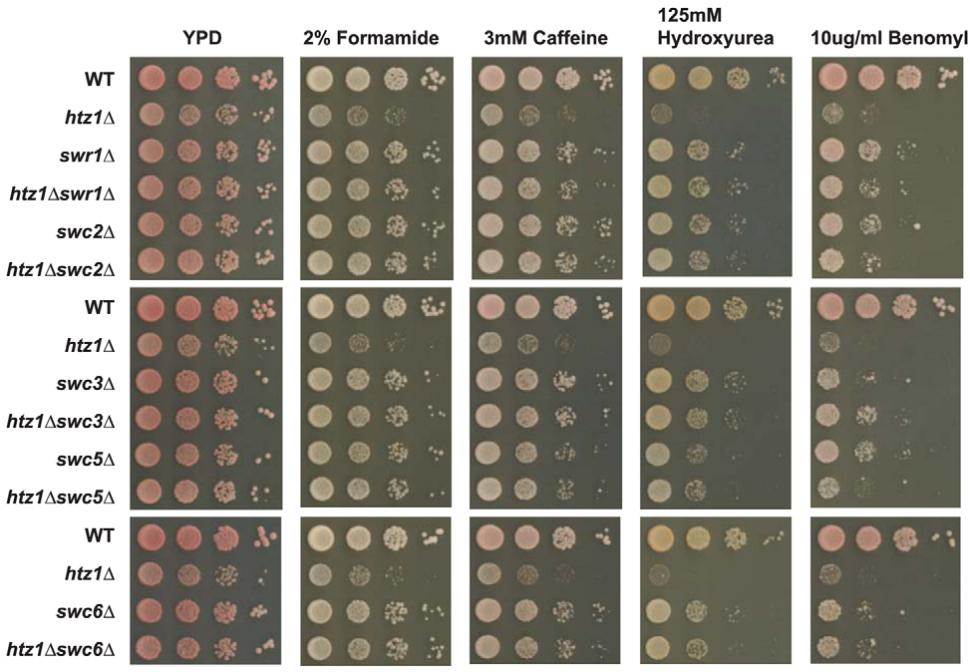
The *htz1*Δ mutant phenotypes were partially suppressible by mutations in genes encoding members of SWR1-Com. The stress sensitivities of *htz1*Δ (MK1027), *swr1*Δ (MKY1028), *htz1*Δ*/swr1*Δ (MKY1029), *swc2*Δ (MKY1030), *htz1*Δ*/swc2*Δ (MKY1031), *swc3*Δ (MKY1032), *htz1*Δ*/swc3*Δ (MKY1033), *swc5*Δ (MKY1034), *htz1*Δ*/swc5*Δ (MKY1035), *swc6*Δ (MKY1036), and *htz1*Δ*/swc6*Δ (MKY1037) strains were assessed by plating ten-fold serial dilutions of these double mutant cultures onto solid YP-glucose (2%) medium with the following conditions: 2% formamide, 3 mM caffeine, 125 mM hydroxyurea, and 10 µg/ml benomyl.

SWR1-Com deposits H2A.Z into chromatin in a two-step process, removing H2A from nucleosomes and subsequently replacing it with H2A.Z [13]. We hypothesized that if H2A.Z were not available, then SWR1-Com might still perform the first step of this mechanism, disrupting the structure of nucleosomes at those positions at which H2A.Z would normally reside, and that this disruption could affect normal promoter function. Thus, the phenotype of cells lacking H2A.Z might be a composite of two different defects: the lack of H2A.Z's function per se, and SWR1-Com's nucleosome-disrupting activity in the absence of H2A.Z. If this hypothesis were correct, then a subset of *htz1*Δ's phenotypes should be suppressed in cells lacking SWR1-Com function. Indeed as predicted by this model, strains with the *htz1*Δ mutation in combination with a null mutation in any gene encoding an important component of the SWR1 complex (*SWR1*, *SWC2*, *SWC3*, *SWC5*, and *SWC6*) exhibited less severe mutant phenotypes than *htz1*Δ single-mutant strains on medium containing compounds that each cause a different type of stress (Figure 7).

**Figure 8:**
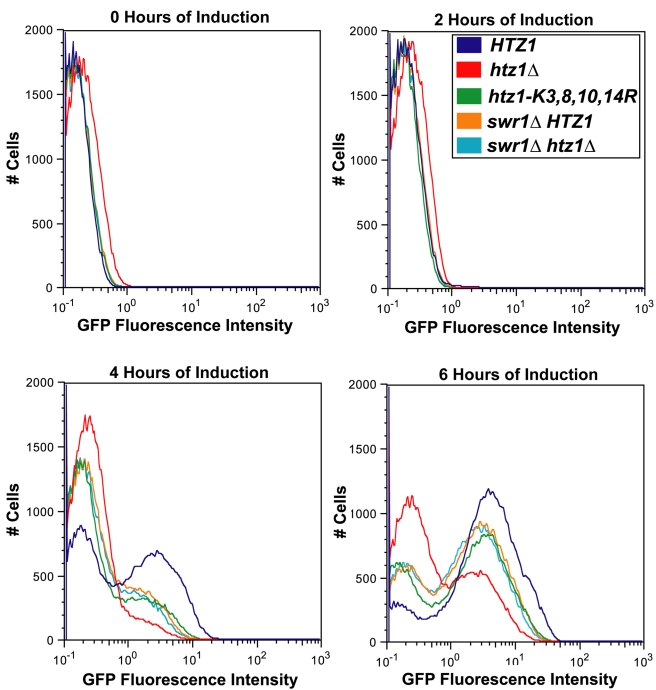
The severity of the *htz1*Δ mutant *GAL1* expression defect was suppressible by the *swr1*Δ mutation. Flow cytometry analysis was performed using Gal1-GFP on *HTZ1* (JRY9002), *htz1*Δ (JRY9004), *htz1-K3,8,10,14R* (JRY9003), *swr1*Δ *HTZ1* (JRY9005), and *swr1*Δ *htz1*Δ (JRY9006) cultures grown long-term in YP-glucose (2%) prior to being transferred into YP-galactose (2%). The histograms represent the distribution of cells within *HTZ1*, *htz1*Δ, and *htz1-K3,8,10,14R* cultures as a function of their GFP intensity.

**Figure 9:**
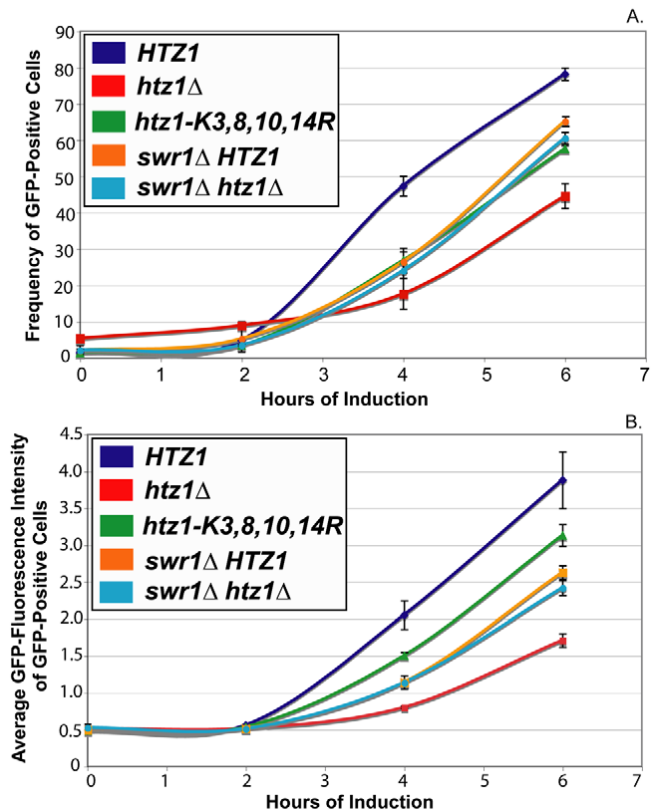
*swr1*Δ mutants' *GAL1* induction phenotypes resembled those of *htz1-K3,8,10,14R* cultures. A threshold level of GFP-intensity was as above. (A) The frequency of GFP positive cells within *HTZ1*, *htz1*Δ, *htz1-K3,8,10,14R*, *swr1*Δ *HTZ1*, and *swr1*Δ *htz1*Δ cultures. (B) The average GFP intensity of the GFP-positive populations of *HTZ1*, *htz1*Δ, *htz1-K3,8,10,14R*, *swr1*Δ *HTZ1*, and *swr1*Δ *htz1*Δ cultures. Bars in all panels represent the standard deviations of values from three biological replicates.

To determine if the *htz1*Δ mutant's galactose-induction was more defective than that of the unacetylatable H2A.Z mutant for a similar reason, the *GAL1* expression phenotypes of both *swr1*Δ *HTZ1* (JRY9005) and *swr1*Δ *htz1*Δ (JRY9006) double-mutant cultures were determined using flow cytometry. Prior to induction, *htz1-K3,8,10,14R*, *swr1*Δ *HTZ1*, and *swr1*Δ *htz1*Δ cultures had similar proportions of GFP-positive cells, and fewer GFP-positive cells than *htz1*Δ cultures (Figures 8 and 9A). Thus, the *swr1*Δ mutation completely suppressed the *htz1*Δ mutant's apparent glucose-repression defect. At every postinduction time point, *swr1*Δ *HTZ1* and *swr1*Δ *htz1*Δ cultures had similar proportions of GFP-positive cells to *htz1-K3,8,10,14R* cultures and significantly higher proportions of GFP-positive cells than *htz1*Δ cultures (Figures 8 and 9A). The *swr1*Δ *HTZ1* and *swr1*Δ *htz1*Δ cells induced *GAL1* expression as rapidly as *htz1-K3,8,10,14R* cells and significantly earlier than *htz1*Δ cells (Table 4). Thus, the severity of the *htz1*Δ mutant's delayed *GAL1*-induction phenotype was suppressible by the *swr1*Δ mutation and therefore likely resulted from the SWR1 complex's activity in the absence of H2A.Z. Furthermore, because *htz1-K3,8,10,14R* cells and *htz1*Δ *swr1*Δ cells needed approximately the same amount of time to induce *GAL1*, H2A.Z's role in promoting rapid *GAL1* activation completely depended on its acetylation.

Interestingly, *GAL1*-expressing cells from *swr1*Δ *HTZ1* and *swr1*Δ *htz1*Δ cultures had significantly higher average GFP intensities than those from *htz1*Δ cultures but they had significantly lower average GFP intensities than those in *htz1-K3,8,10,14R* cultures (Figures 8 and 9B). Quantitative analysis revealed that *GAL1*-expressing cells from both *swr1*Δ *HTZ1* and *swr1*Δ *htz1*Δ cultures accumulated Gal1-GFP 18.8% (+/−5.3%) more rapidly than *htz1*Δ cells and 23.8% (+/−6.7%) more slowly than *htz1-K3,8,10,14R* cells (Table 5). Thus the severity of the *htz1*Δ mutant's Gal1-GFP-accumulation-rate phenotype was suppressible by the *swr1*Δ mutation and therefore likely resulted from the activity of SWR1-Com in H2A.Z's absence. Moreover, our finding that *swr1*Δ *HTZ1* and *swr1*Δ *htz1*Δ cells accumulated Gal1-GFP more slowly than *htz1-K3,8,10,14R* cells suggested that H2A.Z has an acetylation-independent role in increasing *GAL1*-expression rate.

## Discussion

### H2A.Z Was Important for *GAL1* Induction, but Not for Transcriptional Memory

In this work, we showed that H2A.Z, through its acetylation, contributed to induction of the *GAL1* gene, a paradigmatic example of a highly inducible gene of *Saccharomyces*. Acetylated H2A.Z contributed to *GAL1* induction both by increasing the fraction of cells that induced at each time point, and by increasing the level of expression per induced cell. Earlier work established that *GAL1* induction has a property termed transcriptional memory, reflecting the ability of cells that were recently induced to be more easily reinduced following short incubations in repressing conditions than after extended incubations in repressing conditions [27]–[29]. Moreover, H2A.Z has been thought to be a key participant in this transcriptional memory [27]. The conclusion that H2A.Z is important for *GAL1* transcriptional memory is based on experiments involving the induction of *GAL1* as a function of its expression history: when induced from long-term growth in repressing conditions, both *htz1*Δ and *HTZ1* cultures were reported to have induced *GAL1* at similar rates. *htz1*Δ cultures were reported to have induced *GAL1* at a similar rate regardless of whether they had been grown under repressing conditions for either short or long periods of time. However, *HTZ1* cultures that were grown in repressing conditions for short periods of time were reported to induce *GAL1* expression much more rapidly than those grown in repressing conditions for long periods of time [27].

Our work was originally directed at understanding the importance of H2A.Z acetylation to the role of H2A.Z in *GAL1*- transcriptional memory. To this end, we determined the *GAL1*- induction phenotypes of *htz1-K3,8,10,14R* cultures, which carry an unacetylatable allele of H2A.Z. Surprisingly, both *htz1*Δ and *htz1-K3,8,10,14R* cultures grown in inducing conditions prior to short-term growth in repressing conditions induced *GAL1* expression more rapidly than those grown long-term in repressing conditions prior to galactose induction. Thus, both *htz1*Δ and *htz1-K3,8,10,14R* cells exhibited *GAL1* transcriptional memory. Moreover, regardless of whether they were grown long-term or short-term in repressing conditions prior to induction, *htz1*Δ and *htz1-K3,8,10,14R* cultures induced *GAL1* more slowly than *HTZ1* cultures. These results indicated that H2A.Z was important for *GAL1* induction regardless of a cell's growth conditions prior to induction. Thus, the galactose-induction defects that we observed for *htz1*Δ and *htz1-K3,8,10,14R* strains grown short-term in repressing conditions prior to galactose induction were reflective of H2A.Z and acetylated H2A.Z having a general role in *GAL1* induction rather than a specific role in *GAL1*- transcriptional memory.

If H2A.Z or H2A.Z acetylation had a specific role in transcriptional memory, then one would expect cells lacking H2A.Z or containing only an unacetylatable form of H2A.Z to exhibit more severe phenotypes when reinduced than during the primary induction. However, quantitative analysis of the *GAL1* induction phenotypes of the *htz1*Δ and *htz1-K3,8,10,14R* strains indicated that the difference between the two mutant strains' *GAL1* induction phenotypes were less severe, with respect to the *HTZ1* strain's *GAL1* induction phenotypes, when cultures of these strains were reinduced rather than induced. Thus, neither H2A.Z nor acetylated H2A.Z contributed to *GAL1*-transcriptional memory, other than in the general processes of *GAL1* transcription, at least under the conditions of these experiments.

To be completely clear, our data did not discount the existence of what has been referred to as transcriptional memory of *GAL1* induction. A better explanation for memory has been provided by the discovery that the Gal1p protein itself has both galactokinase activity that is crucial for galactose metabolism, as well as Gal3 activity, which is also encoded by the separate *GAL3* gene. Gal3p activates the *GAL4*-encoded activator of *GAL1* induction. Thus *GAL1*-transcriptional memory can be explained by a positive feedback loop in which *GAL1* induction leads to the synthesis of a protein that is both an enzyme and an autoinducer, as shown by others [29],[34]. Our contribution was limited to discounting a role for H2A.Z in this memory.

This understanding of *GAL1*-transcriptional memory suggests a possible explanation for why previously published experiments concluded that *htz1*Δ cultures lack *GAL1*- transcriptional memory [27]. The model presented above posits that a cell's ability to reinduce *GAL1* expression rapidly following short-term repression requires the persistence of Gal1p in the cytoplasm. Thus the amount of time that dividing cells retain the ability to rapidly reinduce *GAL1* expression following repression is a function of both the stability of Gal1p and its abundance prior to glucose repression. The abundance of Gal1p in a cell prior to glucose repression is important because of its dilution with cell division, and thus at some number of cell divisions, the amount of Gal1p will not meet the threshold level required for its role in *GAL1* reinduction. We observed that *htz1*Δ cultures had a nearly 20% lower steady-state *GAL1* expression level than *HTZ1* cultures and that when grown long-term in repressing conditions prior to galactose induction *htz1*Δ cultures did not reach this level of expression until galactose induction had proceeded for more than 14 h. If the previously published experiments did not allow galactose induction to occur for a sufficient period of time, then the *htz1*Δ and *HTZ1* cultures used in these experiments would not be directly comparable with respect to Gal1p levels. Thus cells within *htz1*Δ cultures would be less likely than those in *HTZ1* cultures to have sufficiently high Gal1p levels to allow for the rapid reinduction of *GAL1*.

The discrepancies between the previously published data [27] and those presented here, concerning the role of H2A.Z in primary inductions of *GAL1*, have a straightforward explanation. The conclusion that H2A.Z was not important for primary galactose inductions was based upon *htz1*Δ cells having induced *GAL1* expression less well than *HTZ1* cells after a 2-h induction following short-term growth in repressing conditions, whereas *HTZ1* and *htz1*Δ cells induced *GAL1* equally well following long-term growth in repressing conditions. Our observations were quantitatively similar. However, the critical point is that the magnitude of induction at this early time point was negligible in both *htz1*Δ and *HTZ1* cultures. At all longer periods of galactose induction, *htz1*Δ cells induced *GAL1* expression significantly less well than *HTZ1* cells. We believe the earlier conclusions were based upon inadequate induction periods in some experiments.

The original work implicating H2A.Z in transcriptional memory of *GAL1* also reached the same conclusion for *INO1*. However, the data offered in support of these conclusions are weaker than those offered in support of H2A.Z's role in *GAL1* induction memory. First, these studies fail to establish that *S. cerevisiae* exhibits transcriptional memory of *INO1* in the same way that it exhibits transcriptional memory of *GAL1*. Unlike *GAL1*, cells that are grown short term in repressing conditions prior to induction induce *INO1* more slowly and at lower levels than cells that had been grown long term in repressing conditions prior to induction [27]. Thus, transcriptional memory of *INO1* functions in the opposite way of how it functions in *GAL1* transcription—decreasing rather than increasing a cell's response to inducing conditions. Second, since *INO1*-transcriptional memory results in slower *INO1* reinductions, cells lacking *INO1*-transcriptional memory should induce *INO1* more rapidly than cells that have *INO1*- transcriptional memory. These studies show that *htz1*Δ cells both induce and reinduce *INO1* more slowly than *HTZ1* cells [27]. Therefore, *htz1*Δ cells do not seem to lack transcriptional memory of *INO1*, rather they seem to exhibit defective *INO1* transcription regardless of whether they had recently induced *INO1* expression.

### SWR1-Com Was Deleterious in Cells Lacking H2A.Z

Because SWR1-Com catalyzes a two-step reaction removing H2A from nucleosomes and replacing it with H2A.Z, we considered the possibility that SWR1-Com's function, in the absence of H2A.Z, might leave those nucleosomes normally destined to receive H2A.Z compromised in some way. Thus the overall phenotype of *htz1*Δ would be a composite of those consequences due to the lack of H2A.Z, and those due to uncoupled H2A removal from nucleosomes. Two lines of evidence supported this hypothesis. First, the severity of *htz1*Δ cells' sensitivities to various agents with different mechanisms and targets were substantially suppressible by mutations in genes encoding subunits of SWR1-Com. Second, the difference between *GAL1* induction in *htz1*Δ cells and in cells with unacetylatable H2A.Z was largely suppressed by the *swr1*Δ mutation, creating the less severe phenotype of the unacetylatable H2A.Z mutant. This model is further supported by the observation that *htz1*Δ cells have chromatin that is in the partially open configuration at the *PHO5* promoter under noninducing conditions [21]. We predict that this partially open configuration is a physical manifestation of the mischief wrought by the Swr1-Complex in the absence of H2A.Z.

The benomyl-sensitivity phenotype of the *swc5Δ htz1*Δ double mutant suggests another possible explanation for why SWR1-Com is dangerous for cells that lack H2A.Z. Unlike the *swr1*Δ, *swc2*Δ, *swc3*Δ, and *swc6*Δ mutations that strongly suppressed the *htz1*Δ mutant's benomyl sensitivity phenotype, the *swc5*Δ mutation only weakly suppressed this phenotype. In vitro studies have shown that SWR1-Com complexes lacking Swc2p, Swc6p, Swc4p, Yaf9, or Arp6 bind nucleosomes less well than complete SWR1-Com complexes. In contrast, SWR1-Com complexes that lack Swc5p bind nucleosomes better than complete SWR1-Com complexes [35]. Since Swc5p is required for SWR1-Com's function, the simplest model for why the *swc5*Δ mutation does not strongly suppress the *htz1*Δ mutant's benomyl sensitivity is that mutant SWR1-Com complexes lacking Swc5 may persist in chromatin, perhaps removing H2A, but be unable to replace it with H2A.Z.

### H2A.Z Had Two Distinct Roles in *GAL1* Expression

Our observation that *swr1*Δ*htz1*Δ cells required more time to induce *GAL1* expression, and expressed *GAL1* more slowly once induced, suggested that H2A.Z had two distinct roles in *GAL1* expression—one allowing efficient induction of *GAL1*, and another to increase the rate of *GAL1* expression. That H2A.Z had a role in *GAL1* induction was not surprising given H2A.Z's enrichment at the *GAL1*-promoter. However, that H2A.Z had a role in increasing *GAL1*'s expression rate, as inferred from our model, was unexpected.

There are two lines of evidence that H2A.Z may be important for the expression, per se, of actively transcribed genes. First, even though H2A.Z predominantly localizes to promoters, it is not completely absent from open reading frames (ORFs). The *ACT1* and *PRP8* ORFs, two loci that have been historically considered nonenriched for H2A.Z, are slightly enriched for H2A.Z relative to no-tag controls (Figure S5). Second, the *htz1*Δ mutant is sensitive to 6-azauracil, a toxic compound that slows the growth rate of cells that are defective in mRNA transcript elongation [36]. Thus, it is possible that H2A.Z plays a direct role in transcript elongation. Recent reports raise that possibility further, showing that H2A.Z may aid expression by suppressing antisense transcripts [37].

In summary, our results established that H2A.Z plays no significant role in *GAL1*- transcriptional memory. In contrast H2A.Z, and its acetylation contributed to both the induction of the gene and to its expression per se, adding valuable new insights into one of the best-studied examples of eukaryotic gene regulation. In addition, we showed that SWR1-Com caused defects in gene expression and induction in the absence of H2A.Z, presumably due to nucleosome disruption, that force a reevaluation of all previously described phenotypes of cells lacking H2A.Z.

## Materials and Methods

### Strain Construction

All of the strains used in this study are presented in Table 6. All of these strains were from the W303 background. One-step integration of knockout cassettes has been previously described [38]. JRY9001 was constructed by transforming the *KanMX* cassette into JRY7754. To generate KWY2512, the DNA sequence encoding GFP was inserted before the stop codon of the *GAL1* open reading by transforming a *HIS3*-marked construct encoding the GFP protein. JRY9002, JRY9003, JRY9004 were segregants from crosses of JRY7972, JRY7983, and JRY9001 to KWY2512, respectively. JRY9005 and JRY9006 were segregants from crosses of JRY7752 to JRY9002 and JRY9004, respectively. JRY9011, JRY9012, JRY9013, JRY9014, JRY9015, and JRY9016 were segregants from crosses of JRY9000 to JRY7972, JRY7983, JRY9007, JRY9008, JRY9009, and JRY9010, respectively. MKY1028/MKY1029, MKY1030/MKY1031, MKY1032/MKY1033, MKY1034/MKY1035, and MKY1036/MKY1037 were created by disrupting *SWR1*, *SWC2*, *SWC3*, *SWC5*, and *SWC6* respectively in MKY1038 using a Sp*HIS5MX* knockout cassette that was amplified from pFA6a-His3MX6 [38].

**Table 6:** Yeast strains used in this study.

Strain	Genotype	Source
W303-1a	*MAT*α *ade2-1 leu2-3 112 his3-1 ura3-52 trp1-100 can1-100*	R. Rothstein
CRY1	*MATa ade2–1 can1–100 his3–11,15 leu2–3,112 trp1–1 ura3–1*	[27]
DBY50	*MAT*a *htz1*Δ*::His5 ade2–1 can1–100 his3–11,15 leu2–3,112 trp1–1 ura3–1 SEC63-13myc::Kan' INO1:LacO128:URA3 HIS3:LacI-GFP MAT*	[27]
KWY2512	*MATa GAL1-GFP::HIS3MX*	This study
JRY7752	*MAT*α *swr1*Δ*::SpHIS5MX*	[14]
JRY7754	*MAT*α *htz1*Δ*::SpHIS5MX*	[14]
JRY7970	*MAT*α *htz1*Δ*::URA3MX*	[17]
JRY7972	*MAT*α *HTZ1-3Flag::KanMX*	[17]
JRY7983	*MAT*α *htz1K3,8,10,14R-3Flag::KanMX*	[17]
JRY9000	*MATa htz1*Δ*::URA3MX GAL1-GFP::HIS3MX ADE2*	This study
JRY9001	*MAT*α *htz1*Δ*::KanMX*	This study
JRY9002	*MATa HTZ1-3Flag::KanMX GAL1-GFP::HIS3MX ADE2*	This study
JRY9003	*MATa htz1-K3,8,10,14R-3Flag::KanMX GAL1-GFP::HIS3MX ADE2*	This study
JRY9004	*MATa htz1*Δ*::KanMX GAL1-GFP::HIS3MX ADE2*	This study
JRY9005	*MATa swr1*Δ*::SpHIS5MX HTZ1-3Flag::KanMX GAL1-GFP::HIS3MX ADE2*	This study
JRY9006	*MATa swr1*Δ*::SpHIS5MX htz1::KanMX GAL1-GFP::HIS3MX ADE2*	This study
JRY9007	*MAT*α *htz1-K3R-3Flag::KanMX*	[17]
JRY9008	*MATα htz1-K8R-3Flag::KanMX*	[17]
JRY9009	*MAT*α *htz1-K10R-3Flag::KanMX*	[17]
JRY9010	*MAT*α *htz1-K14R-3Flag::KanMX*	[17]
JRY9011	*MATa/MAT*α *HTZ1-3Flag::KanMX/htz1*Δ*::caURA3 GAL1-GFP::HIS3MX/GAL1 ADE2/ade2-1*	This study
JRY9012	*MATa/MAT*α *htz1-K3,8,10,14R3Flag:: KanMX/htz1*Δ*:: caURA3 GAL1-GFP::HIS3MX/GAL1 ADE2/ade2-1*	This study
JRY9013	*MATa/MAT*α *htz1-K3R-3Flag::KanMX/htz1*Δ*::caURA3 GAL1-GFP::HIS3MX/GAL1 ADE2/ade2-1*	This study
JRY9014	*MATa/MAT*α *htz1-K8R-3Flag::KanMX/htz1*Δ*::caURA3 GAL1-GFP::HIS3MX/GAL1 ADE2/ade2-1*	This study
JRY9015	*MATa/MAT*α *htz1-K10-3FlagR::KanMX/htz1*Δ*::caURA3 GAL1-GFP::HIS3MX/GAL1 ADE2/ade2-1*	This study
JRY9016	*MATa/MAT*α *htz1-K14R-3Flag::KanMX/htz1*Δ*::caURA3 GAL1-GFP::HIS3MX/GAL1 ADE2/ade2-1*	This study
MKY1027	*MATa htz1*Δ*::KanMX*	This study
MKY1028	*MATa swr1*Δ*::SpHIS5MX*	This study
MKY1029	*MATa htz1*Δ*::KanMX swr1*Δ*::SpHIS5MX*	This study
MKY1030	*MATa swc2*Δ*::SpHIS5MX*	This study
MKY1031	*MATa htz1*Δ*::KanMX swc2*Δ*::SpHIS5MX*	This study
MKY1032	*MATa swc3*Δ*::HIS5MX*	This study
MKY1033	*MATa htz1*Δ*::KanMX swc3*Δ*::SpHIS5MX*	This study
MKY1034	*MATa swc5*Δ*::SpHIS5MX*	This study
MKY1035	*MATa htz1*Δ*::KanMX swc5*Δ*::SpHIS5MX*	This study
MKY1036	*MATa swc6*Δ*::SpHIS5MX*	This study
MKY1037	*MATa htz1*Δ*::KanMX swc6*Δ*::SpHIS5MX*	This study
MKY1052	*MATa*/*MAT*α htz1Δ::KanMX/HTZ1	This study

### Culturing of Yeast

Yeast media were as defined [39]. Seed culture density affected *GAL1* induction phenotypes, so precautions were taken to ensure that seed cultures of all strains had similar growth histories. Specifically, seed cultures for all experiments were grown in YP-Dextrose (D-glucose, 2%) except DBY50 and CRY1, which were grown in CSM-Dextrose (D-glucose, 2%). 50 ml seed cultures were inoculated with cells from a single colony and grown overnight with shaking at 30°C to OD 0.2, and were then harvested by centrifugation at 2,060*g* for 1 min. The cells were then washed with 25 ml of prewarmed 30°C YP-galactose and resuspended in 50 ml of 30°C YP-galactose, except in experiments performed with DBY50 and CRY1, in which CSM-Galactose was used instead of YP-galactose for both washing and resuspending in order to follow precisely the procedures of others [27]. The volume of culture removed for each time point was replaced with the same volume of 30°C YP-galactose.

### RNA Analysis and ChIP

Both determination of mRNA levels by quantitative reverse-transcriptase (Q-RT) PCR and ChIP were performed as described [17] except that SYBR GreenER (Invitrogen) PCR reagents were used. H2A.Z-3Flag, and H2A.Z-K3,8,10,14R-3Flag were immunoprecipitated using the αFlag M2 resin (Sigma).

### Flow Cytometry

Cells were harvested by centrifugation, fixed in a 4% paraformaldehyde/3.4% sucrose solution for 10 min at room temperature and then stored overnight at 4°C in a 1.2 M sorbitol solution with KPO_4_ buffer at pH 7.5. GFP expression data were collected for each sample using the FC-500 (Beckman-Coulter) flow cytometer and analyzed using the Flow-Jo software package. The *GAL1*-GFP expression status of individual cells within cultures on a cell-by-cell basis in each culture was determined by plotting flow-cytometry measurements as a histogram of GFP fluorescence (*y*-axis number of cells; *x*-axis Log GFP intensity relative to GFP-negative values). The threshold of GFP intensity was set so that between 1% and 2% of glucose-grown *HTZ1* cultures would be classified as GFP-positive. Cells that had GFP-intensity greater than this threshold value were counted as GFP positive (*GAL1-GFP* expressing). The level of *GAL1* expression in different populations was calculated by determining the geometric mean GFP intensity.

### Quantitative Analysis of mRNA Expression Levels

We developed a simple mathematical model to analyze the dynamics of *GAL1* mRNA expression levels. This model allowed us to robustly quantify the onset time of *GAL1* induction, steady state *GAL1* mRNA level, and the time needed to reach half of the steady-state level. The model is based on three parameters, which we optimized to maximize the fit of the model to the measured *GAL1* mRNA levels. These include: (1) the time *x* when induction of *GAL1* mRNA begins; (2) the rate α at which *GAL1* mRNA is produced; and (3) the rate δ at which GAL1 mRNA molecules are being degraded.

According to the model, the relative amount of *GAL1* mRNA at time *t*, *M(t)*, follows the ordinary differential equation (ODE):
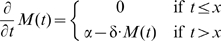
Namely, *GAL1* is not being expressed at all until time point *x*, from which point it is produced at a fixed rate *α*, and being degraded at a fixed ratio δ, until it reaches the steady state equilibrium:
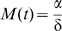
Given the model parameters, and starting from zero *M*(0) = 0, we can solve the ordinary differential equation using the Runge-Kutta method (as implemented in MATLAB 7.6), and estimate the mRNA level of *GAL1* at every time point *t*.

We optimized the three parameters *x*, α, and δ for every culture to minimize the root-mean-square deviation (RMSD) between the experimental measurements and the modeled values. The values that were used in each of the best-fit models are presented in Table S1. We constrained the parameters *x*, α, and δ to non-negative values, and used the active-set optimization algorithm (FMINCON function in MATLAB 7.6). For the memory experiments, the optimized values of the *GAL1* expression onset times, for all cultures, were very close to zero, and practically below the time resolution of the model and data. We therefore simplified the model, and explicitly set *x* to zero.

Finally, to estimate the half steady-state time point, we used the optimized parameters for each culture to find the steady state level α/δ, and to solve the ordinary differential equation and identify when *GAL1* levels reach half of the steady state level.

### Quantitative Modeling of Flow Cytometry Data

To analyze the flow cytometry data, the time-course measurements of single-cell Gal1-GFPp intensities were transformed into *GAL1-GFP* induction times and Gal1-GFP accumulation rates. To do this, a simplified model of *GAL1*-induction was developed, and its six parameters fitted to the measured data for each culture. For every cell, this model assumes that *GAL1* is completely repressed until its induction time *t*_i_, when cellular Gal1-GFPp begins to accumulate at a fixed rate *x*_i_. We therefore model *E*_i_(t), the Gal1-GPFp content of the *i*th cell at time *t* as:
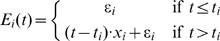
where:

- The induction time of the *i*th cell, *t*_i_, is sampled from a Gamma distribution with parameters (*k*_t_,θ_t_)
- The Gal1-GFPp accumulation rate of the *i*th cell, *x*_i_, is independently sampled from a Gamma distribution with parameters (*k*_x_,θ_x_)

The estimated expression is added to a stochastic noise term ε_i_, drawn from a Normal distribution with parameters (μ,σ^2^), to simulate a basal level of *GAL1* expression.

The model was used to simulate a population of 100,000 cells, whose *GAL1*-*GFP*-induction times *t*_i_'s and accumulation rates *x*_i_'s were sampled independently from two Gamma distributions: *t*_i_ ∼ Gamma (k_t_,θ_t_), and *x*_i_ ∼ Gamma (k_x_,θ_x_), and their stochastic noise terms sampled from a Normal distribution: *ε_i_* ∼ Normal(μ,σ^2^). Given a set of six parameters (*k*_t_, θ_t_, *k*_x_, θ_x_, μ, σ^2^) this model sampled activation times, accumulation rates, and noise terms for each of the 100,000 cells in the simulation, and computed the cellular Gal1-GFPp levels *E*_i_(t) for each of the four times points that were measured (0, 2, 4, and 6 h following induction), which allowed for the simulation of flow-cytometry outputs. Activation times and accumulation rates were sampled from a stochastic distribution rather than being fixed at specific values to account for the natural variability among cells because of biological variables like cell size, position in the cell cycle, cell age, and other factors that were not treated as variables in the model. Gamma distributions were used due to their non-negativity property.

The parameters of the model were optimized by minimizing the root-mean-squared deviation between the measured data (average of triplicates) and the model predictions, summed over the four measured time points (0, 2, 4, and 6 h.) To optimize these parameters, genetic algorithms were used (as implemented in the GA function in MATLAB 7.6) followed by a derivative-free optimization using the simplex algorithm (FMINSEARCH function in MATLAB 7.6). These optimization steps were repeated with 200 random starting points for each strain, and the optimal set of parameters were then selected (Tables S2 and S3). The error in our estimation of each strain's induction time and accumulation rate was calculated by determining the range of values for each parameter that were used in the top 50 best-fit simulations for each strain.

The models that were determined for each strain's Gal1-GFP expression phenotype were used as a proxy to quantitatively compare the *GAL1*-activation times and Gal1-GFPp accumulation rates of *HTZ1*, *htz1-K3,8,10,14R*, *htz1*Δ, *swr1*Δ *HTZ1* and *swr1*Δ *htz1*Δ cells.

## Supporting Information

Figure S1 **The distribution of** ***GAL1*** **-induction times and Gal-GFPp accumulation rates among cells from** ***htz1***Δ** cultures as modeled as a gamma distribution of values.** See text for details. (A) shows the Gamma distribution of *GAL1*-induction times that were used in the best-fit simulations of *htz1*Δ *GAL1-GFP* expression phenotype. (B) shows the Gamma distribution of Gal1-GFP accumulation rates that were used in the best-fit simulations of *htz1*Δ *GAL1-GFP* expression phenotype. (C) compares the *GAL1-GFP* induction phenotypes that were observed for *htz1*Δ cultures with the phenotype that was predicted for each culture based on its best-fit simulation.
(0.81 MB TIF)

Figure S2 **The distribution of** ***GAL1*** **-induction times and Gal-GFPp accumulation rates among cells from** ***htz1-K3,8,10,14R*** **cultures as modeled as a gamma distribution of values.** See text for details. (A) shows the Gamma distribution of *GAL1*-induction times that were used in the best-fit simulations of *htz1-K3,8,10,14R GAL1-GFP* expression phenotype. (B) shows the Gamma distribution of Gal1-GFP accumulation rates that were used in the best-fit simulations of *htz1-K3,8,10,14R GAL1-GFP* expression phenotype. (C) compares the *GAL1-GFP* induction phenotypes that were observed for *htz1-K3,8,10,14R* cultures with the phenotype that was predicted for each culture based on its best-fit simulation.
(0.81 MB TIF)

Figure S3 **The distribution of** ***GAL1*** **-induction times and Gal-GFPp accumulation rates among cells from** ***swr1*** **Δ** ***HTZ1*** **cultures as modeled as a gamma distribution of values.** See text for details. (A) shows the Gamma distribution of *GAL1*-induction times that were used in the best-fit simulations of *swr1*Δ *HTZ1 GAL1-GFP* expression phenotype. (B) shows the Gamma distribution of Gal1-GFP accumulation rates that were used in the best-fit simulations of *swr1*Δ *HTZ1 GAL1-GFP* expression phenotype. (C) compares the *GAL1-GFP* induction phenotypes that were observed for *swr1*Δ *HTZ1* cultures with the phenotype that was predicted for each culture based on its best-fit simulation.
(0.81 MB TIF)

Figure S4 **The distribution of** ***GAL1*** **-induction times and Gal-GFPp accumulation rates among cells from** ***swr1*** **Δ** ***htz1*** **Δ cultures as modeled as a gamma distribution of values.** See text for details. (A) shows the Gamma distribution of *GAL1*-induction times that were used in the best-fit simulations of *swr1*Δ *htz1*Δ *GAL1-GFP* expression phenotype. (B) shows the Gamma distribution of Gal1-GFP accumulation rates that were used in the best-fit simulations of *swr1*Δ *htz1*Δ *GAL1-GFP* expression phenotype. (C) compares the *GAL1-GFP* induction phenotypes that were observed for *swr1*Δ *htz1*Δ cultures with the phenotype that was predicted for each culture based on its best-fit simulation.
(0.80 MB TIF)

Figure S5 **H2A.Z localizes to the ORFs of** ***ACT1*** **and** ***PRP8*** **.** ChIP analysis of H2A.Z-FLAG enrichment at the *ACT1* and *PRP8* ORFs in *HTZ1-Flag* (JRY7972) cultures that were grown long-term in YP-glucose (2%). Bars represent the standard deviation of three biological replicates.
(0.06 MB TIF)

Figure S6 **Measurements of Gal1-GFP accumulation by flow cytometry were reproducible.** Flow cytometry analysis was performed using Gal1-GFP on *HTZ1* (JRY9002) cells grown long-term in YP-glucose (2%) prior to being transferred into YP-galactose (2%). The histograms in this figure represent the distribution of cells within each culture as a function of their GFP intensity. The individual FACS plots of three biological replicates are shown for *HTZ1*.
(0.62 MB TIF)

Figure S7 **Measurements of Gal1-GFP accumulation by flow cytometry were reproducible.** Flow cytometry analysis was performed using Gal1-GFP on *htz1*Δ (JRY9004) cells grown long-term in YP-glucose (2%) prior to being transferred into YP-galactose (2%). The histograms in this figure represent the distribution of cells within each culture as a function of their GFP intensity. The individual FACS plots of three biological replicates are shown for *htz1*Δ.
(0.53 MB TIF)

Figure S8 **Measurements of Gal1-GFP accumulation by flow cytometry were reproducible.** Flow cytometry analysis was performed using Gal1-GFP on *htz1-K3,8,10,14R* (JRY9003) cells grown long-term in YP-glucose (2%) prior to being transferred into YP-galactose (2%). The histograms in this figure represent the distribution of cells within each culture as a function of their GFP intensity. The individual FACS plots of three biological replicates are shown for *htz1-K3,8,10,14R*.
(0.65 MB TIF)

Table S1 **Parameters used in mathematical model of** ***GAL1*** **mRNA data.**
(0.07 MB DOC)

Table S2 **Parameters used in mathematical model of** ***GAL1*** **activation times.**
(0.06 MB DOC)

Table S3 **Parameters used in mathematical model of** ***GAL1*** **expression rates.**
(0.06 MB DOC)

## Abbreviations

ORF: open reading frame
GFP: green fluorescent protein

## References

[1] Ozdemir A, Spicuglia S, Lasonder E, Vermeulen M, Campsteijn C (2005). Characterization of lysine 56 of histone H3 as an acetylation site in Saccharomyces cerevisiae.. J Biol Chem.

[2] Hassan A. H, Prochasson P, Neely K. E, Galasinski S. C, Chandy M (2002). Function and selectivity of bromodomains in anchoring chromatin-modifying complexes to promoter nucleosomes.. Cell.

[3] Lachner M, O'Carroll D, Rea S, Mechtler K, Jenuwein T (2001). Methylation of histone H3 lysine 9 creates a binding site for HP1 proteins.. Nature.

[4] Pray-Grant M. G, Daniel J. A, Schieltz D, Yates J. R, Grant P. A (2005). Chd1 chromodomain links histone H3 methylation with SAGA- and SLIK-dependent acetylation.. Nature.

[5] Ruthenburg A. J, Allis C. D, Wysocka J (2007). Methylation of lysine 4 on histone H3: intricacy of writing and reading a single epigenetic mark.. Mol Cell.

[6] Strahl B. D, Allis C. D (2000). The language of covalent histone modifications.. Nature.

[7] Taverna S. D, Ilin S, Rogers R. S, Tanny J. C, Lavender H (2006). Yng1 PHD finger binding to H3 trimethylated at K4 promotes NuA3 HAT activity at K14 of H3 and transcription at a subset of targeted ORFs.. Mol Cell.

[8] Meluh P. B, Yang P, Glowczewski L, Koshland D, Smith M. M (1998). Cse4p is a component of the core centromere of Saccharomyces cerevisiae.. Cell.

[9] Guillemette B, Bataille A. R, Gevry N, Adam M, Blanchette M (2005). Variant histone H2A.Z is globally localized to the promoters of inactive yeast genes and regulates nucleosome positioning.. PLoS Biol.

[10] Zhang H, Roberts D. N, Cairns B. R (2005). Genome-wide dynamics of Htz1, a histone H2A variant that poises repressed/basal promoters for activation through histone loss.. Cell.

[11] Li B, Pattenden S. G, Lee D, Gutierrez J, Chen J (2005). Preferential occupancy of histone variant H2AZ at inactive promoters influences local histone modifications and chromatin remodeling.. Proc Natl Acad Sci U S A.

[12] Raisner R. M, Hartley P. D, Meneghini M. D, Bao M. Z, Liu C. L (2005). Histone variant H2A.Z marks the 5′ ends of both active and inactive genes in euchromatin.. Cell.

[13] Mizuguchi G, Shen X, Landry J, Wu W. H, Sen S (2004). ATP-driven exchange of histone H2AZ variant catalyzed by SWR1 chromatin remodeling complex.. Science.

[14] Kobor M. S, Venkatasubrahmanyam S, Meneghini M. D, Gin J. W, Jennings J. L (2004). A protein complex containing the conserved Swi2/Snf2-related ATPase Swr1p deposits histone variant H2A.Z into euchromatin.. PLoS Biol.

[15] Krogan N. J, Keogh M. C, Datta N, Sawa C, Ryan O. W (2003). A Snf2 family ATPase complex required for recruitment of the histone H2A variant Htz1.. Mol Cell.

[16] Meneghini M. D, Wu M, Madhani H. D (2003). Conserved histone variant H2A.Z protects euchromatin from the ectopic spread of silent heterochromatin.. Cell.

[17] Babiarz J. E, Halley J. E, Rine J (2006). Telomeric heterochromatin boundaries require NuA4-dependent acetylation of histone variant H2A.Z in *Saccharomyces cerevisiae*.. Genes Dev.

[18] Keogh M. C, Mennella T. A, Sawa C, Berthelet S, Krogan N. J (2006). The *Saccharomyces cerevisiae* histone H2A variant Htz1 is acetylated by NuA4.. Genes Dev.

[19] Millar C. B, Xu F, Zhang K, Grunstein M (2006). Acetylation of H2AZ Lys 14 is associated with genome-wide gene activity in yeast.. Genes Dev.

[20] Wan Y, Saleem R. A, Ratushny A. V, Roda O, Smith J. J (2009). Role of the histone variant H2A.Z/Htz1p in TBP recruitment, chromatin dynamics, and regulated expression of oleate-responsive genes.. Mol Cell Biol.

[21] Santisteban M. S, Kalashnikova T, Smith M. M (2000). Histone H2A.Z regulates transcription and is partially redundant with nucleosome remodeling complexes.. Cell.

[22] Adam M, Robert F, Larochelle M, Gaudreau L (2001). H2A.Z is required for global chromatin integrity and for recruitment of RNA polymerase II under specific conditions.. Mol Cell Biol.

[23] Adams B. G (1972). Induction of galactokinase in Saccharomyces cerevisiae: kinetics of induction and glucose effects.. J Bacteriol.

[24] Douglas H. C, Hawthorne D. C (1964). Enzymatic expression and genetic linkage of genes controlling galactose utilization in Saccharomyces.. Genetics.

[25] Hashimoto H, Kikuchi Y, Nogi Y, Fukasawa T (1983). Regulation of expression of the galactose gene cluster in Saccharomyces cerevisiae. Isolation and characterization of the regulatory gene *GAL4*.. Mol Gen Genet.

[26] Hopper J. E, Rowe L. B (1978). Molecular expression and regulation of the galactose pathway genes in Saccharomyces cerevisiae. Distinct messenger RNAs specified by the *Gal*1 and *Gal7* genes in the *Gal7*-*Gal10*-*Gal1* cluster.. J Biol Chem.

[27] Brickner D. G, Cajigas I, Fondufe-Mittendorf Y, Ahmed S, Lee P. C (2007). H2A.Z-mediated localization of genes at the nuclear periphery confers epigenetic memory of previous transcriptional state.. PLoS Biol.

[28] Kundu S, Horn P. J, Peterson C. L (2007). SWI/SNF is required for transcriptional memory at the yeast GAL gene cluster.. Genes Dev.

[29] Zacharioudakis I, Gligoris T, Tzamarias D (2007). A yeast catabolic enzyme controls transcriptional memory.. Curr Biol.

[30] Lemieux K, Larochelle M, Gaudreau L (2008). Variant histone H2A.Z, but not the HMG proteins Nhp6a/b, is essential for the recruitment of Swi/Snf, Mediator, and SAGA to the yeast *GAL1* UAS(G).. Biochem Biophys Res Commun.

[31] Gligoris T, Thireos G, Tzamarias D (2007). The Tup1 corepressor directs Htz1 deposition at a specific promoter nucleosome marking the *GAL1* gene for rapid activation.. Mol Cell Biol.

[32] Biggar S. R, Crabtree G. R (2001). Cell signaling can direct either binary or graded transcriptional responses.. Embo J.

[33] Ren Q, Gorovsky M. A (2001). Histone H2A.Z acetylation modulates an essential charge patch.. Mol Cell.

[34] Acar M, Becskei A, van Oudenaarden A (2005). Enhancement of cellular memory by reducing stochastic transitions.. Nature.

[35] Wu W. H, Alami S, Luk E, Wu C. H, Sen S (2005). Swc2 is a widely conserved H2AZ-binding module essential for ATP-dependent histone exchange.. Nat Struct Mol Biol.

[36] Desmoucelles C, Pinson B, Saint-Marc C, Daignan-Fornier B (2002). Screening the yeast “disruptome” for mutants affecting resistance to the immunosuppressive drug, mycophenolic acid.. J Biol Chem.

[37] Zofall M, Fischer T, Zhang K, Zhou M, Cui B (2009). Histone H2A.Z cooperates with RNAi and heterochromatin factors to suppress antisense RNAs.. Nature.

[38] Longtine M. S, McKenzie A, Demarini D. J, Shah N. G, Wach A (1998). Additional modules for versatile and economical PCR-based gene deletion and modification in Saccharomyces cerevisiae.. Yeast.

[39] Amberg D. C, Burke D. J, Strathern J. N (2005). Methods in yeast genetics: a Cold Spring Harbor Laboratory course manual.

