# Supplementary figures and images for: Roles for H2A.Z and Its Acetylation in GAL1 Transcription and Gene Induction, but Not GAL1-Transcriptional Memory
Source: PLoS Biol. 2010 Jun 22;8(6):e1000401. doi: 10.1371/journal.pbio.1000401 (PMC2889906; doi:10.1371/journal.pbio.1000401)

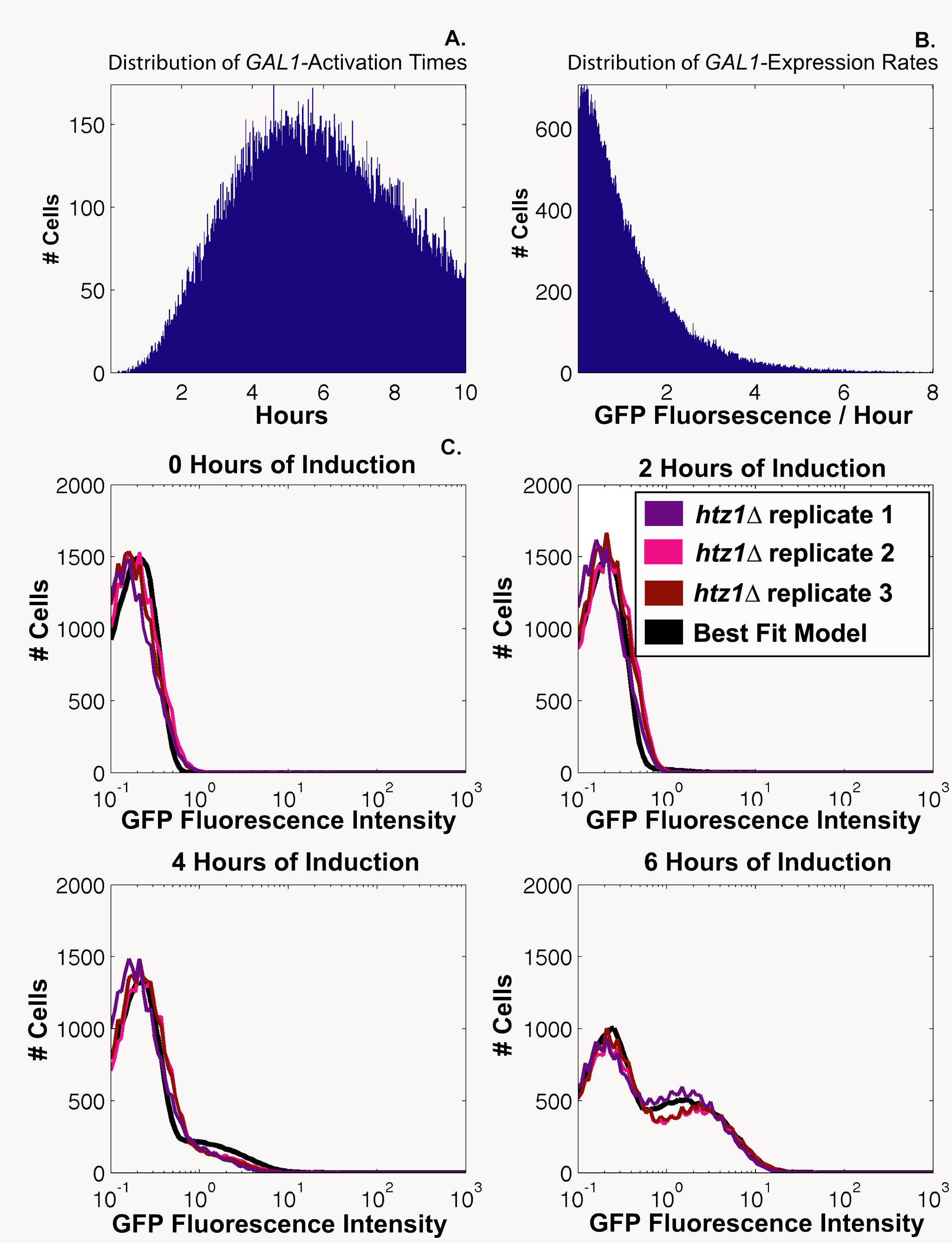

Supplement: Figure S1 — The distribution of GAL1 -induction times and Gal-GFPp accumulation rates among cells from htz1Δ cultures as modeled as a gamma distribution of values. See text for details. (A) shows the Gamma distribution of GAL1-induction times that were used in the best-fit simulations of htz1Δ GAL1-GFP expression phenotype. (B) shows the Gamma distribution of Gal1-GFP accumulation rates that were used in the best-fit simulations of htz1Δ GAL1-GFP expression phenotype. (C) compares the GAL1-GFP induction phenotypes that were observed for htz1Δ cultures with the phenotype that was predicted for each culture based on its best-fit simulation. (0.81 MB TIF) [file pbio.1000401.s001.tif]

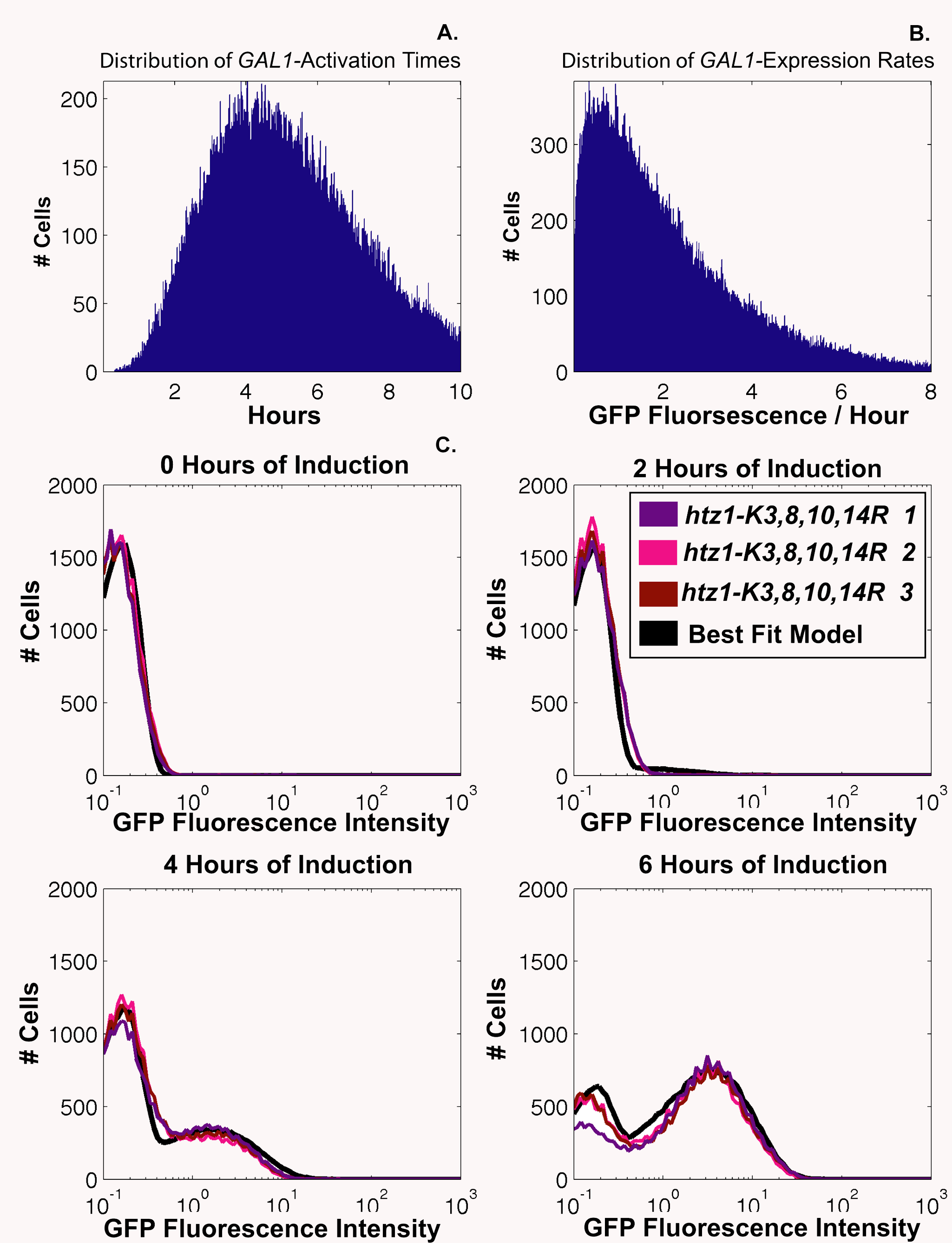

Supplement: Figure S2 — The distribution of GAL1 -induction times and Gal-GFPp accumulation rates among cells from htz1-K3,8,10,14R cultures as modeled as a gamma distribution of values. See text for details. (A) shows the Gamma distribution of GAL1-induction times that were used in the best-fit simulations of htz1-K3,8,10,14R GAL1-GFP expression phenotype. (B) shows the Gamma distribution of Gal1-GFP accumulation rates that were used in the best-fit simulations of htz1-K3,8,10,14R GAL1-GFP expression phenotype. (C) compares the GAL1-GFP induction phenotypes that were observed for htz1-K3,8,10,14R cultures with the phenotype that was predicted for each culture based on its best-fit simulation. (0.81 MB TIF) [file pbio.1000401.s002.tif]

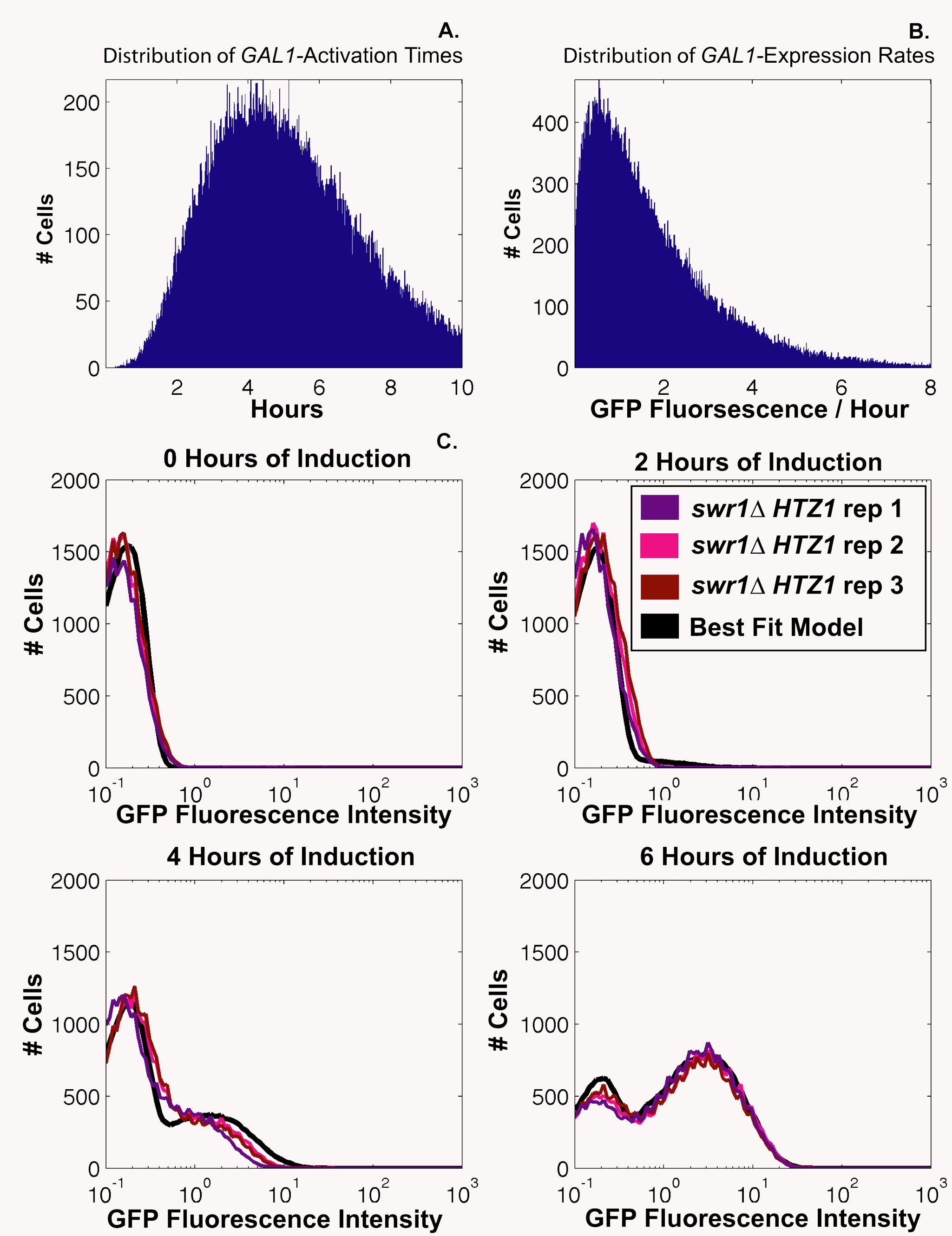

Supplement: Figure S3 — The distribution of GAL1 -induction times and Gal-GFPp accumulation rates among cells from swr1 Δ HTZ1 cultures as modeled as a gamma distribution of values. See text for details. (A) shows the Gamma distribution of GAL1-induction times that were used in the best-fit simulations of swr1Δ HTZ1 GAL1-GFP expression phenotype. (B) shows the Gamma distribution of Gal1-GFP accumulation rates that were used in the best-fit simulations of swr1Δ HTZ1 GAL1-GFP expression phenotype. (C) compares the GAL1-GFP induction phenotypes that were observed for swr1Δ HTZ1 cultures with the phenotype that was predicted for each culture based on its best-fit simulation. (0.81 MB TIF) [file pbio.1000401.s003.tif]

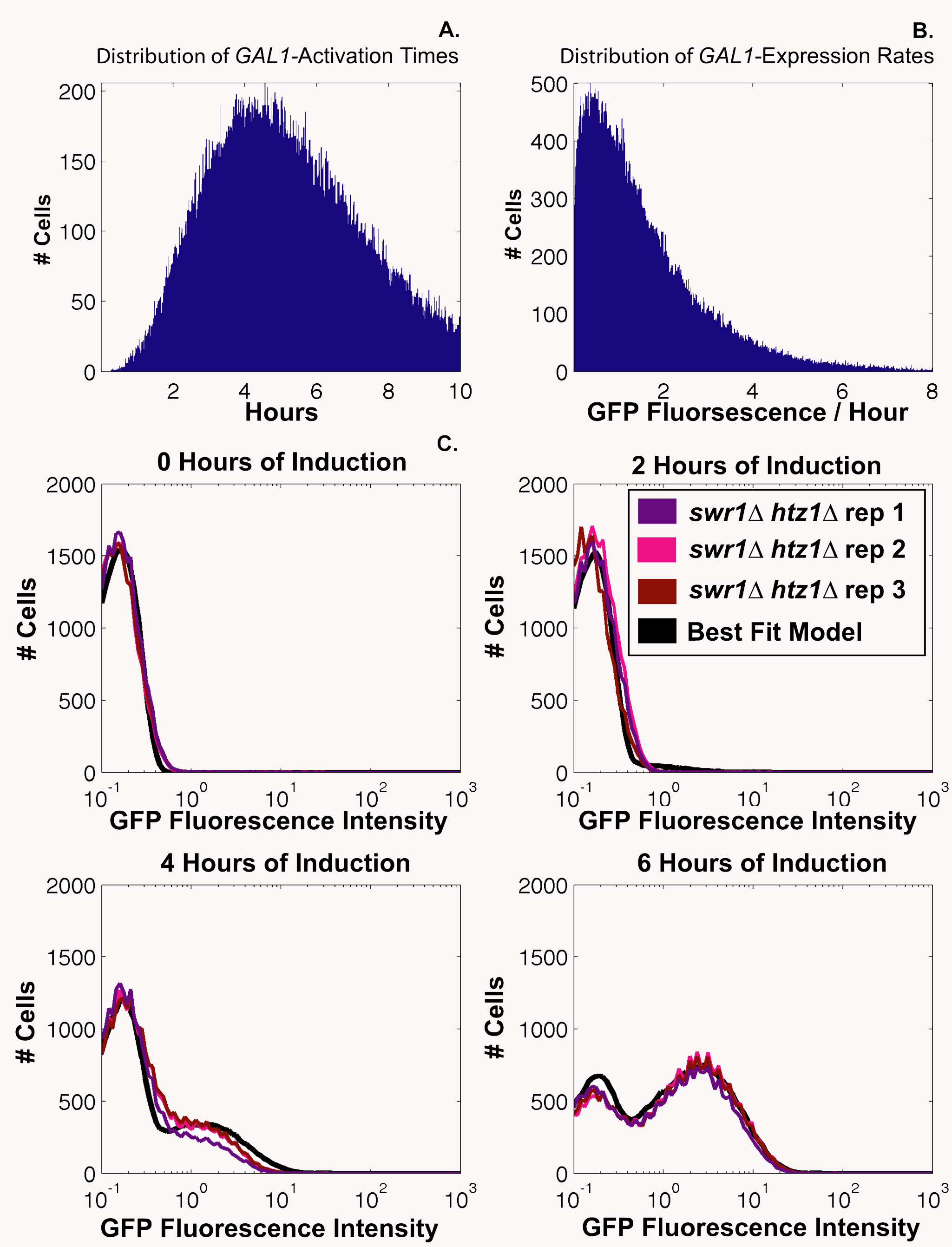

Supplement: Figure S4 — The distribution of GAL1 -induction times and Gal-GFPp accumulation rates among cells from swr1 Δ htz1 Δ cultures as modeled as a gamma distribution of values. See text for details. (A) shows the Gamma distribution of GAL1-induction times that were used in the best-fit simulations of swr1Δ htz1Δ GAL1-GFP expression phenotype. (B) shows the Gamma distribution of Gal1-GFP accumulation rates that were used in the best-fit simulations of swr1Δ htz1Δ GAL1-GFP expression phenotype. (C) compares the GAL1-GFP induction phenotypes that were observed for swr1Δ htz1Δ cultures with the phenotype that was predicted for each culture based on its best-fit simulation. (0.80 MB TIF) [file pbio.1000401.s004.tif]

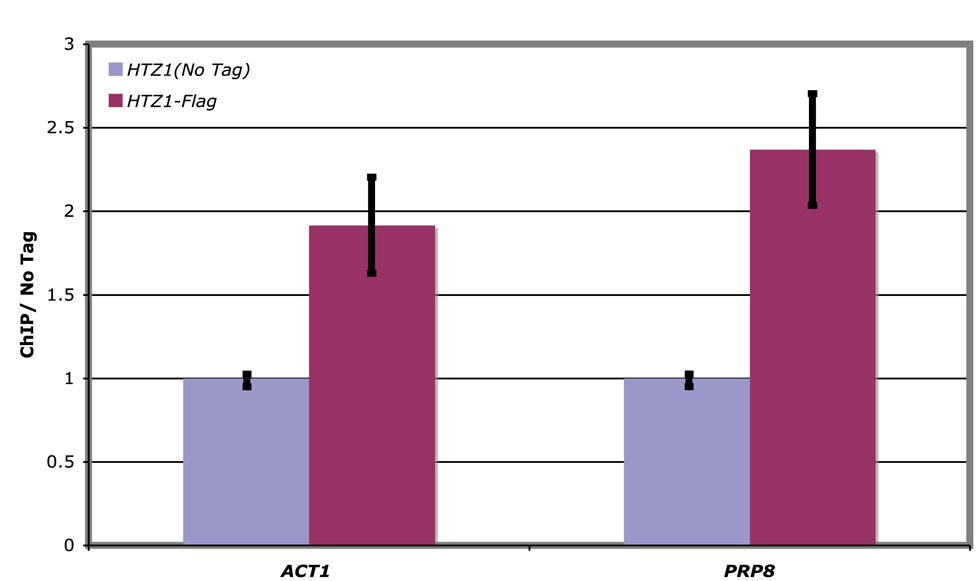

Supplement: Figure S5 — H2A.Z localizes to the ORFs of ACT1 and PRP8 . ChIP analysis of H2A.Z-FLAG enrichment at the ACT1 and PRP8 ORFs in HTZ1-Flag (JRY7972) cultures that were grown long-term in YP-glucose (2%). Bars represent the standard deviation of three biological replicates. (0.06 MB TIF) [file pbio.1000401.s005.tif]

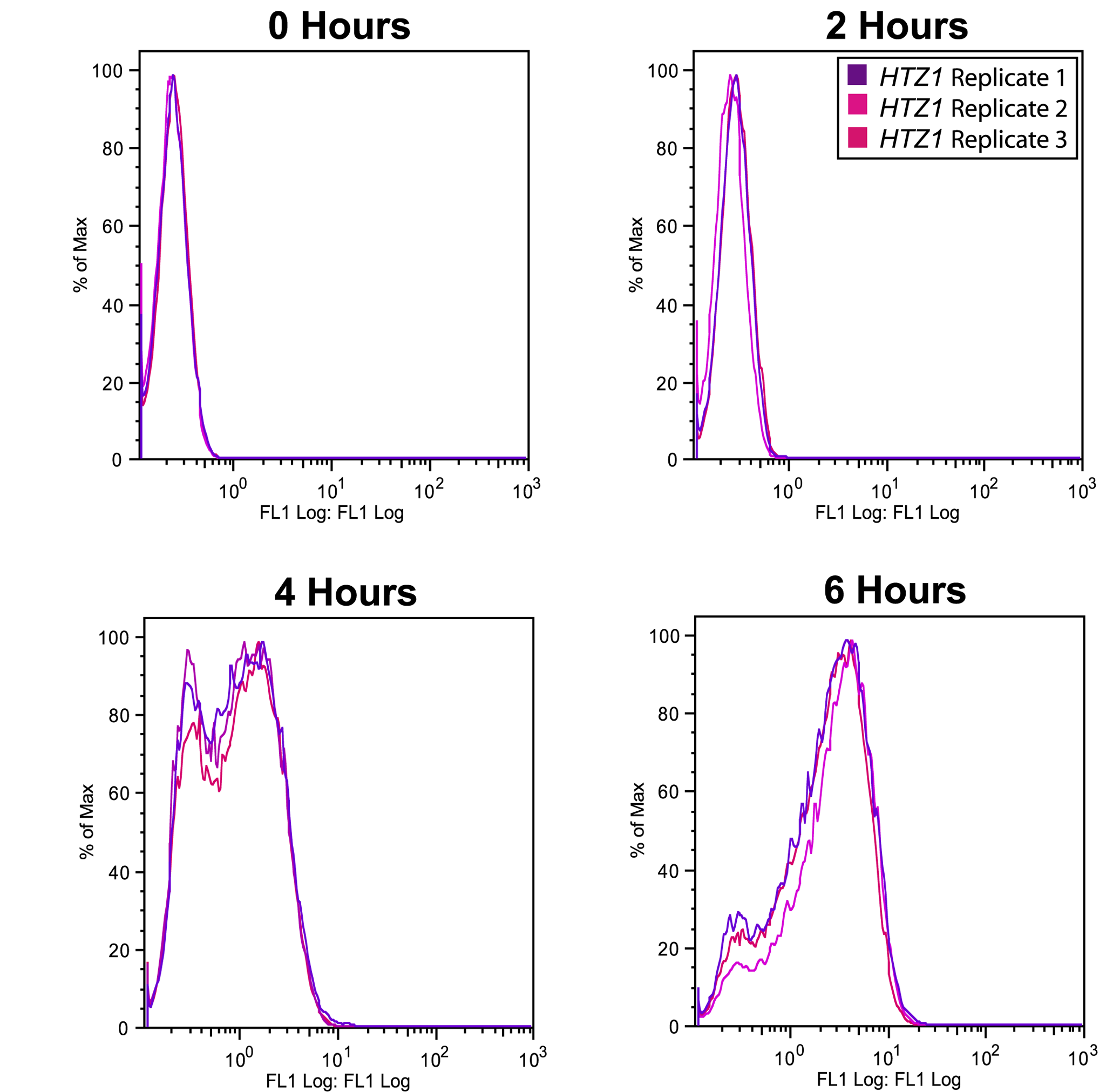

Supplement: Figure S6 — Measurements of Gal1-GFP accumulation by flow cytometry were reproducible. Flow cytometry analysis was performed using Gal1-GFP on HTZ1 (JRY9002) cells grown long-term in YP-glucose (2%) prior to being transferred into YP-galactose (2%). The histograms in this figure represent the distribution of cells within each culture as a function of their GFP intensity. The individual FACS plots of three biological replicates are shown for HTZ1. (0.62 MB TIF) [file pbio.1000401.s006.tif]

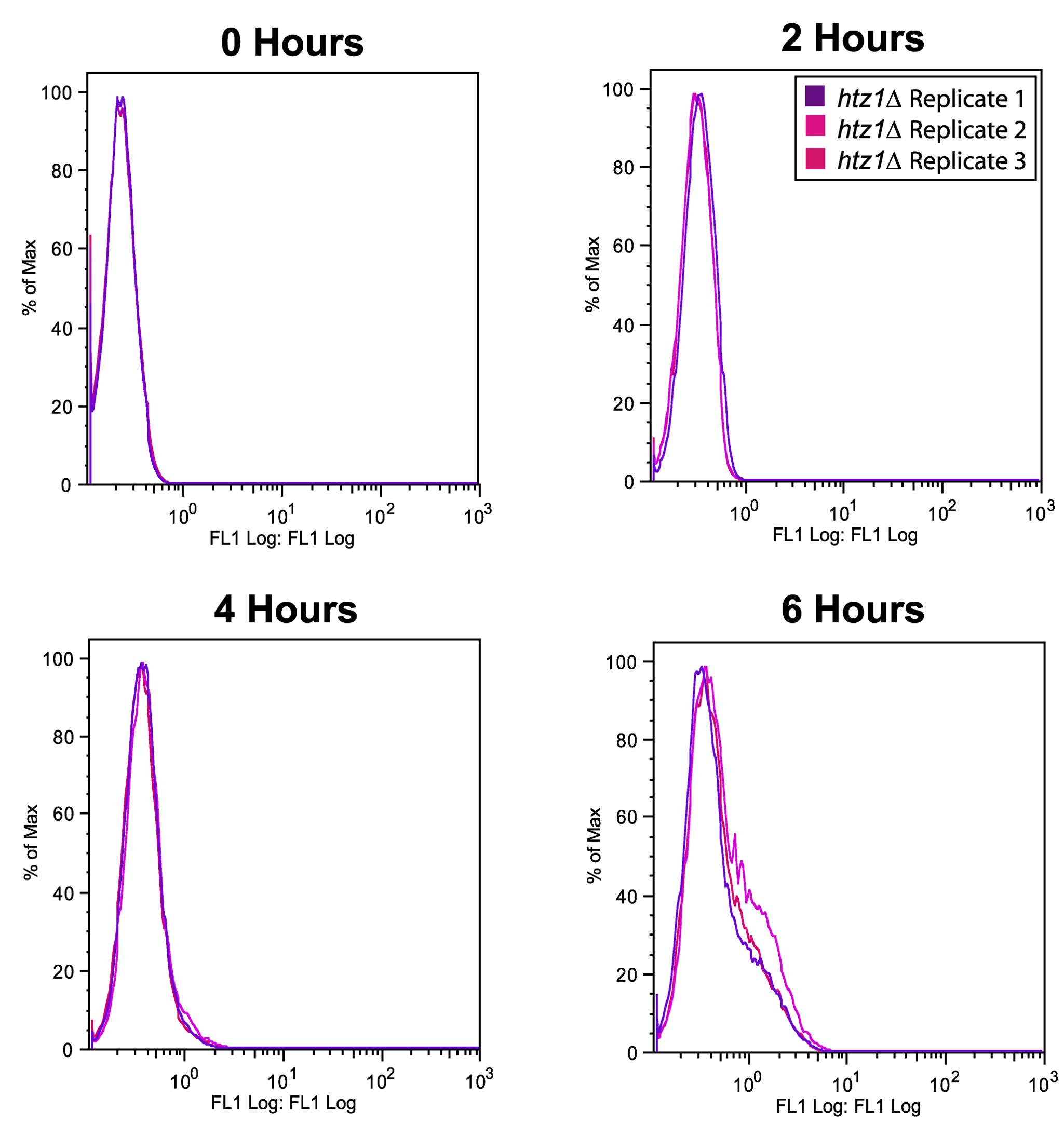

Supplement: Figure S7 — Measurements of Gal1-GFP accumulation by flow cytometry were reproducible. Flow cytometry analysis was performed using Gal1-GFP on htz1Δ (JRY9004) cells grown long-term in YP-glucose (2%) prior to being transferred into YP-galactose (2%). The histograms in this figure represent the distribution of cells within each culture as a function of their GFP intensity. The individual FACS plots of three biological replicates are shown for htz1Δ. (0.53 MB TIF) [file pbio.1000401.s007.tif]

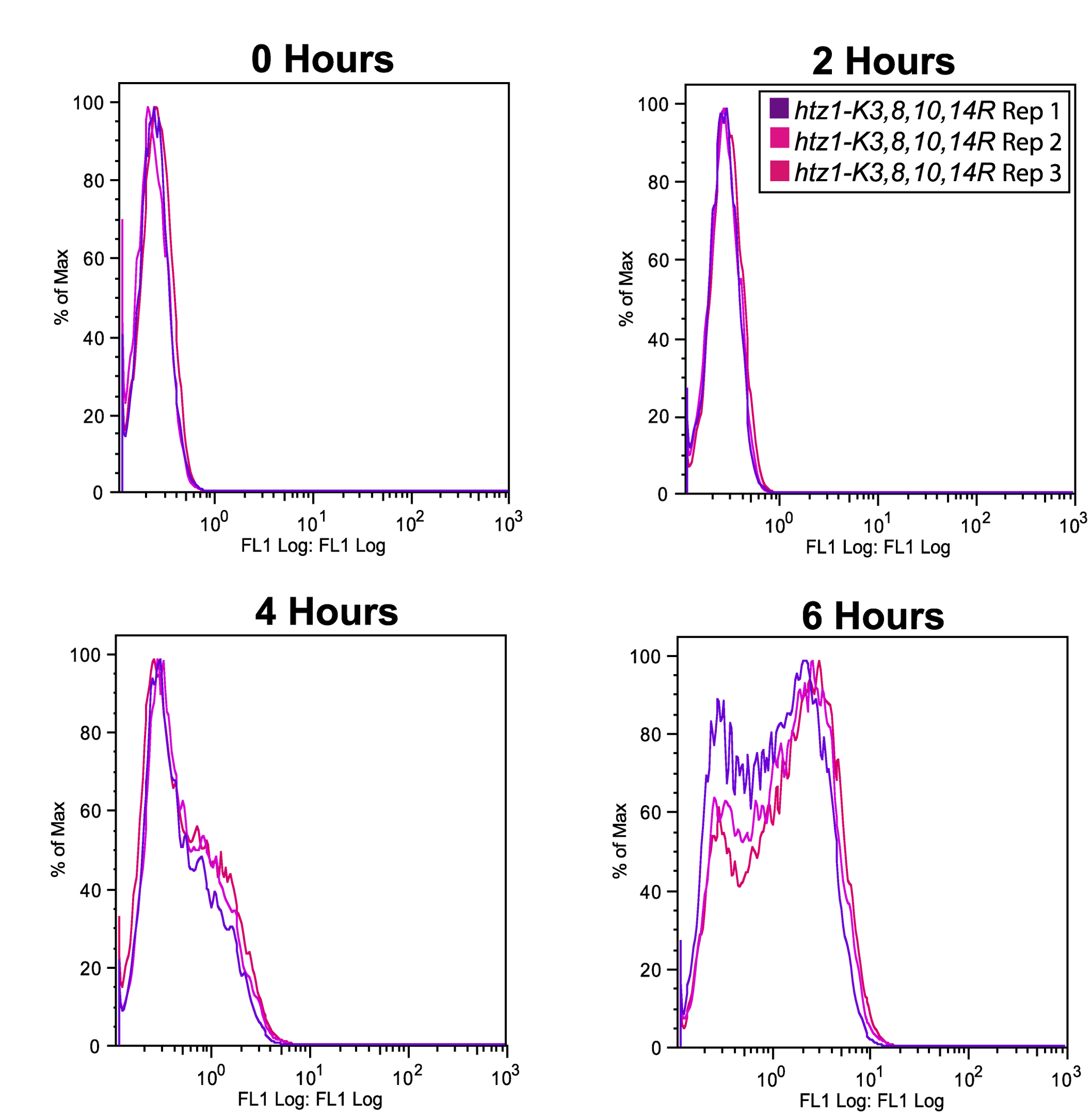

Supplement: Figure S8 — Measurements of Gal1-GFP accumulation by flow cytometry were reproducible. Flow cytometry analysis was performed using Gal1-GFP on htz1-K3,8,10,14R (JRY9003) cells grown long-term in YP-glucose (2%) prior to being transferred into YP-galactose (2%). The histograms in this figure represent the distribution of cells within each culture as a function of their GFP intensity. The individual FACS plots of three biological replicates are shown for htz1-K3,8,10,14R. (0.65 MB TIF) [file pbio.1000401.s008.tif]
