# Supplementary material for: Roles for H2A.Z and Its Acetylation in GAL1 Transcription and Gene Induction, but Not GAL1-Transcriptional Memory
Source: PLoS Biol. 2010 Jun 22;8(6):e1000401. doi: 10.1371/journal.pbio.1000401 (PMC2889906; doi:10.1371/journal.pbio.1000401)
Supplement: Table S1 — Parameters used in mathematical model of GAL1 mRNA data. (0.07 MB DOC) [file pbio.1000401.s009.doc]

**Supplementary Table S1: Parameters Used in Mathematical Model of *GAL1* mRNA Data**

|  | **Column** | **A** | **B** | **C** | **D** | **E** | **F** | **G** | **H** | **I** | **J** |
| --- | --- | --- | --- | --- | --- | --- | --- | --- | --- | --- | --- |
|  | **Strain** | **Primary Induction *GAL1* Transcription Activation Time (Minutes)** | **Secondary Induction *GAL1* Transcription Activation Time (Minutes)** | **Primary Induction *GAL1* Accumulation Rate (*GAL1*/*ACT1*/Minute)** | **Secondary Induction *GAL1* Accumulation Rate (*GAL1*/*ACT1*/Minute)** | **Primary Induction *GAL1* Degradation Rate**  **(*GAL1*/*ACT1*/Minute)** | **Secondary Induction *GAL1* Degradation Rate**  **(*GAL1*/*ACT1*/Minute)** | **Primary Induction *GAL1* Steady State Expression Level (*GAL1/ACT1*)** | **Secondary Induction *GAL1* Steady State Expression Level (*GAL1/ACT1*)** | **Primary Induction Time to Half-Steady-State *GAL1* Expression Level (Minutes)** | **Secondary Induction Time to Half-Steady-State *GAL1* Expression Level (Minutes)** |
|  | ***HTZ1*** | 204.8 | 0* | 0.022 | 0.044 | 0.024 | 0.047 | 0.91 | 0.94 | 234.3 | 14.8 |
|  | ***htz1∆*** | 214.1 | 0* | 0.003 | 0.010 | 0.005 | 0.018 | 0.67 | 0.55 | 362.4 | 38.9 |
|  | ***htz1-K3,8,10,14R*** | 191.3 | 0* | 0.003 | 0.017 | 0.004 | 0.023 | 0.77 | 0.74 | 375.4 | 29.7 |
|  | ***HTZ1* (CRY1)** | 204.4 | 0* | 0.014 | 0.024 | 0.018 | 0.024 | 0.84 | 0.97 | 243.7 | 28.4 |
|  | ***htz1∆***  **(DBY 50)** | 218.9 | 0* | 0.003 | 0.004 | 0.005 | 0.006 | 0.67 | 0.71 | 356.3 | 110.4 |

*The data suggested that the time to first induction of *GAL1* for all strains in the secondary induction­­ experiments was very close to zero, and could not be distinguished from it.
