# Supplementary material for: Roles for H2A.Z and Its Acetylation in GAL1 Transcription and Gene Induction, but Not GAL1-Transcriptional Memory
Source: PLoS Biol. 2010 Jun 22;8(6):e1000401. doi: 10.1371/journal.pbio.1000401 (PMC2889906; doi:10.1371/journal.pbio.1000401)
Supplement: Table S2 — Parameters used in mathematical model of GAL1 activation times. (0.06 MB DOC) [file pbio.1000401.s010.doc]

**Supplementary Table S2: Parameters Used in Mathematical Model of *GAL1* Activation Times**

M**ean Time ΓDistribution**

**of *GAL1-*Activation Time By Percentile**  Parameters

| **Strain** | **Activation (Hours)** | **5%** | **10%** | **25%** | **50%** | **75%** | **90%** | **95%** | ***k*** | **θ** |
| --- | --- | --- | --- | --- | --- | --- | --- | --- | --- | --- |
| *HTZ1* | 4.185 | 1.591 | 1.981 | 2.777 | 3.893 | 5.276 | 6.766 | 7.775 | 4.712 | 0.888 |
| *htz1∆* | 6.469 | 2.751 | 3.337 | 4.503 | 6.097 | 8.032 | 10.083 | 11.458 | 5.732 | 1.129 |
| *htz1-k3,8,10,14R* | 5.49 | 2.201 | 2.707 | 3.726 | 5.139 | 6.873 | 8.727 | 9.976 | 5.153 | 1.065 |
| *swr1∆ HTZ1* | 5.24 | 2.107 | 2.589 | 3.561 | 4.907 | 6.558 | 8.323 | 9.511 | 5.178 | 1.012 |
| *swr1∆ htz1∆* | 5.339 | 2.149 | 2.64 | 3.63 | 5 | 6.681 | 8.477 | 9.687 | 5.186 | 1.029 |
