# Supplementary material for: Roles for H2A.Z and Its Acetylation in GAL1 Transcription and Gene Induction, but Not GAL1-Transcriptional Memory
Source: PLoS Biol. 2010 Jun 22;8(6):e1000401. doi: 10.1371/journal.pbio.1000401 (PMC2889906; doi:10.1371/journal.pbio.1000401)
Supplement: Table S3 — Parameters used in mathematical model of GAL1 expression rates. (0.06 MB DOC) [file pbio.1000401.s011.doc]

**Supplementary Table S3: Parameters Used in Mathematical Model of *GAL1* Expression Rates**

**Mean Expression ΓDistribution**

**Rate *GAL1-*GFP Accumulation Rate By Percentile**  Parameters

| **Strain** | **(GFP Counts / Hour)** | **5%** | **10%** | **25%** | **50%** | **75%** | **90%** | **95%** | ***k*** | **θ** |
| --- | --- | --- | --- | --- | --- | --- | --- | --- | --- | --- |
| *HTZ1* | 2.155 | 0.231 | 0.392 | 0.839 | 1.676 | 2.959 | 4.55 | 5.716 | 1.422 | 1.516 |
| *htz1∆* | 1.318 | 0.068 | 0.139 | 0.38 | 0.914 | 1.827 | 3.033 | 3.945 | 1.002 | 1.315 |
| *htz1-k3,8,10,14R* | 2.103 | 0.28 | 0.45 | 0.895 | 1.69 | 2.868 | 4.299 | 5.336 | 1.623 | 1.296 |
| *swr1∆ HTZ1* | 1.637 | 0.179 | 0.302 | 0.642 | 1.277 | 2.247 | 3.448 | 4.327 | 1.437 | 1.14 |
| *swr1∆ htz1∆* | 1.592 | 0.129 | 0.236 | 0.553 | 1.186 | 2.198 | 3.484 | 4.439 | 1.227 | 1.297 |
